# Free Rather Than Total Iron Content Is Critically Linked to the Fur Physiology in *Shewanella oneidensis*

**DOI:** 10.3389/fmicb.2020.593246

**Published:** 2020-11-26

**Authors:** Lulu Liu, Xue Feng, Wei Wang, Yining Chen, Zhe Chen, Haichun Gao

**Affiliations:** Institute of Microbiology, College of Life Sciences, Zhejiang University, Hangzhou, China

**Keywords:** iron homeostasis, *Shewanella*, Fur regulation, heme, oxidative stress

## Abstract

Ferric uptake regulator (Fur) is a transcriptional regulator playing a central role in iron homeostasis of many bacteria, and Fur inactivation commonly results in pleiotropic phenotypes. In *Shewanella oneidensis*, a representative of dissimilatory metal-reducing γ-proteobacteria capable of respiring a variety of chemicals as electron acceptors (EAs), Fur loss substantially impairs respiration. However, to date the mechanism underlying the physiological phenomenon remains obscure. This investigation reveals that Fur loss compromises activity of iron proteins requiring biosynthetic processes for their iron cofactors, heme in particular. We then show that *S. oneidensis* Fur is critical for maintaining heme homeostasis by affecting both its biosynthesis and decomposition of the molecule. Intriguingly, the abundance of iron-containing proteins controlled by H_2_O_2_-responding regulator OxyR increases in the *fur* mutant because the Fur loss activates OxyR. By comparing suppression of membrane-impermeable, membrane-permeable, and intracellular-only iron chelators on heme deficiency and elevated H_2_O_2_ resistance, our data suggest that the elevation of the free iron content by the Fur loss is likely to be the predominant factor for the Fur physiology. Overall, these results provide circumstantial evidence that Fur inactivation disturbs bacterial iron homeostasis by altering transcription of its regulon members, through which many physiological processes, such as respiration and oxidative stress response, are transformed.

## Introduction

Iron is a critical nutrient for virtually all living organisms to survive and grow as it is the most common redox active metal found in proteins (Andrews et al., 2003). Naturally, facultatively anaerobic Gram-negative bacteria, such as *Escherichia coli*, possess hundreds of iron-containing proteins, which participate in an array of biological processes as electron carriers, enzymes, and sensors of environmental and intracellular cues to regulate gene expression (O’Brian, 2015). To facilitate realization of these functions, iron is typically present within heme and iron-sulfur (Fe-S) groups, although there are proteins with iron as mono- and bi-nuclear iron centers (Liu et al., 2014; Rokob et al., 2016). Because of high demand and low solubility of iron in aerobic environments, iron acquisition has always been a challenge to cells, causing iron deficiency to be one of the most common nutritional stresses (Frawley and Fang, 2014). Meanwhile, when overloaded, iron could be highly toxic to cells by efficiently catalyzing biomolecular damages to DNA, proteins, and lipids via Fenton reaction (Imlay, 2013). Given that both iron deficiency and overload can be deleterious, it is imperative that cells maintain proper iron homeostasis (Andrews et al., 2003).

In many bacteria, cellular iron homeostasis is primarily regulated by ferric uptake regulator (Fur), which mediates transcription of a cohort of genes in response to iron availability (Fillat, 2014; Seo et al., 2014). Fur mostly works in the classical model of transcriptional repression, based on the extensively studied *E. coli* paradigm, using ferrous iron (Fe^2+^) as co-repressor (Andrews et al., 2003; Sheikh and Taylor, 2009). When iron is plentiful, Fur interacts with Fe^2+^ to form Fur-Fe^2+^ complexes, which bind to *cis*-acting regulatory sequences known as iron-responsive elements (Fur boxes) to block transcription of operons that follow. Under iron depletion conditions, Fur becomes inactive because of the lack of the metal co-repressor, and derepression occurs (Andrews et al., 2003).

It is well established that repression of iron acquisition genes is the main and highly conserved physiological function of Fur (Fillat, 2014; Seo et al., 2014). Derepression of the Fur regulon by iron depletion (starvation) should result in increased iron uptake, facilitating normal iron homeostasis to be maintained. By logic, Fur inactivation would allow unchecked iron uptake, which eventually leads to iron overload. In some bacteria, this indeed is the case (Sheikh and Taylor, 2009; Jittawuttipoka et al., 2010; Vajrala et al., 2011; Becerra et al., 2014). However, iron deficiency resulting from *fur* mutations has also been reported (Keyer and Imlay, 1996; Kitphati et al., 2007; Qi et al., 2012). Moreover, when Fur is inactivated, some bacteria, such as *E. coli*, suffer from a decrease in the total iron content while facing an increase in the free (unbound) iron content simultaneously (Keyer and Imlay, 1996; Abdul-Tehrani et al., 1999). This has been attributed, at least in part, to constitutive and repressive expression of iron transport and iron storage proteins, respectively. As a consequence, iron uptake and consumption (mainly iron storage) are improperly balanced such that free iron becomes excessive (Andrews et al., 2003).

*Shewanella*, a group of facultative dissimilatory metal-reducing γ-proteobacteria, have attracted interest for their ability to reduce a wide array of electron acceptors (EAs), and they now serve as a genetic model for studying microbial electrosynthesis (Fredrickson et al., 2008; Rabaey and Rozendal, 2010; Lemaire et al., 2020). The respiratory versatility is attributable to a large repertoire of iron-containing proteins, including hundreds having heme, Fe-S clusters as cofactors, and a dozen or so using iron atom directly to execute catalysis (Imlay, 2014). Among them, heme-containing cytochromes *c* (cyts *c*), in which heme *b* molecules are covalently attached to polypeptides (Kranz et al., 2009), play a central role in respiration and are unusually abundant: at least 40 cyts *c* are predicted to be encoded in the genome (Meyer et al., 2004; Gao et al., 2010; Fu et al., 2015). Apparently, the unusually high abundance of iron-containing proteins is a signature of iron-reducing microorganisms because *Geobacter* species, another group of bacteria renowned for exceptional metal-reducing capacity, contain even a larger number of cyts *c* (Methé et al., 2003; Lovley et al., 2011). In line with this, *Shewanella*, as observed in the genus representative *S. oneidensis*, and *Geobacter* have iron content that is significantly higher than model bacteria, such as *E. coli* (Senko and Stolz, 2001; Daly et al., 2004). Because of this, it is conceivable that iron homeostasis plays a particularly important role in endowing metal-reducing bacteria their attractive characteristics.

The importance of iron homeostasis in respiration of both soluble and insoluble EAs in *Shewanella* and *Geobacter* has been known for about two decades, and in the former Fur has been identified and suggested to play an critical role in regulation of these processes (Senko and Stolz, 2001; Thompson et al., 2002; Supplementary Figure S1). Subsequent investigations soon established the relationship between iron homeostasis and Fur activity (Wan et al., 2004; O’Neil et al., 2008; Yang et al., 2008). In *Shewanella*, the loss of Fur results in impaired reduction of a variety of EAs, supporting the proposal that Fur plays a general and critical role in respiration (Yang et al., 2008, 2013; Fu et al., 2018). Although Yang et al. (2013) showed that some of reductase genes are under the direct control of Fur, this level of regulation is unlikely to be a determining factor because the forced production of these genes does not suppress the respective defects observed in the *fur* mutants (Fu et al., 2018). Instead, the respiratory defects of the *fur* mutants are largely due to the compromised overall cyt *c* biosynthesis, presumably a result of reduced heme biosynthesis (Yang et al., 2013; Fu et al., 2018). Given that the Fur loss distorts normal iron homeostasis by reducing total iron while increasing free iron content (Fu et al., 2018), it is reasonable to hypothesize that impaired cyt *c* biosynthesis (reduced heme content) is a consequence of the imbalanced iron homeostasis.

Here in *S. oneidensis*, we set out to investigate the influence of Fur loss on various iron-containing proteins, to unravel the mechanism for the reduced heme levels, and to solve inconsistencies observed before. Our data demonstrate that the core of the Fur physiological roles is to prevent the overload of free iron, which is a result of altered transcription of many Fur regulon members upon Fur inactivation. Once free iron levels reach over a threshold, a variety of biological processes are affected and pleiotropic effects of Fur inactivation emerge.

## Materials and Methods

### Bacterial Strains, Plasmids, and Culture Conditions

The bacterial strains and plasmids used in this study were listed in Table 1. Sequences of the primers used in this study were available on request. All chemicals were obtained from Sigma-Aldrich Co., unless otherwise noted. *E. coli* and *S. oneidensis* were grown aerobically in LB (Difco, Detroit, MI, United States) at 37 and 30°C for genetic manipulation. When appropriate, the growth medium was supplemented with the following: 2, 6-diaminopimelic acid (DAP), 0.3 mM; ampicillin, 50 μg/ml; kanamycin, 50 μg/ml; gentamicin, 15 μg/ml.

**TABLE 1:** Strains and plasmids used in this study.

Strain or plasmid	Description	Source/references
***E. coli* strain**		
DH5α	Host strain for routine cloning	Lab stock
WM3064	Donor strain for conjugation; Δ*dapA*	W. Metcalf, UIUC^a^
***S. oneidensis* strain**		
MR-1	Wild type	ATCC 700550
HG0266	Δ*ccmF* derived from MR-1	Jin et al., 2013
HG1070	Δ*katB* derived from MR-1	Jiang et al., 2014
HG1328	Δ*oxyR* derived from MR-1	Jiang et al., 2014
HG1783-4	Δ*feo* derived from MR-1	Liu et al., 2018
HG1937	Δ*fur* derived from MR-1	Gao et al., 2015
HG3030	Δ*putA* derived from MR-1	Dong et al., 2017
HGPUB	Δ*pubABC* derived from MR-1	Dong et al., 2017
ΔputAΔfeo	Δ*putA*Δ*feo* derived from MR-1	Liu et al., 2014
HG1111-2	Δ*bfr*(Δ*bfr2*Δ*bfr1*) derived from MR-1	Fu et al., 2018
HGCYD	Δ*cyd* derived from MR-1	Chen et al., 2015
Δ*oxyR*Δ*fur*	Δ*oxyR*Δ*fur* derived from MR-1	This study
HG3669-7	Δ*hmuAXZ* derived from MR-1	This study
Δ*hmu*Δ*fur*	Δ*fur*Δ*hmuAXZ* derived from MR-1	This study
Δ*fur*Δ*feo*	Δ*fur*Δ*feo* derived from MR-1	This study
Δ*fur*Δ*pub*	Δ*fur*Δ*pubABC* derived from MR-1	This study
Δ*fur*Δ*bfr*	Δ*fur*Δ*bfr* derived from MR-1	This study
Δ*ccmF*Δ*fur*	Δ*ccmF*Δ*fur* derived from MR-1	This study
Δ*ccmF*Δ*fur*Δ*hmu*	Δ*ccmF*Δ*fur*Δ*hmuAXZ* derived from MR-1	This study
Δ*putA*Δ*feo*Δ*hmu*	Δ*putA*Δ*feo*Δ*hmu* derived from MR-1	This study
**Plasmid**		
pHGM01	Ap^r^ Gm^r^ Cm^r^ suicide vector	Jin et al., 2013
pHGEN-P*tac*	IPTG-inducible P*tac* expression vector	Meng et al., 2018a
pHGE-P*tac*-*fur*	Vector for inducible expression of *fur*	Fu et al., 2018
pHGE-P*tac*-*bfr*	Vector for inducible expression of *bfr*	Fu et al., 2018
pHGE-P*tac*-*oxyR*	Vector for inducible expression of *oxyR*	Wan et al., 2018
pHGEN-P*tac*-*hemA*	Vector for inducible expression of *hemA*	This study
pHGEN-P*tac*-*hmu*	Vector for inducible expression of *hmuAXZ*	This study
pHGEN-P*tac*-*dpa*	Vector for inducible expression of *B. subtilis dpaAB*	This study
pHGEN-P*tac*-*dpa*	Vector for inducible expression of *B. subtilis dpaAB*	This study

*^*a*^UIUC, University of Illinois at Urbana-Champaign.*

For physiological characterization, both LB and defined medium MS (containing Fe(III) [FeCI_3_] at 3.6 μM) supplemented with 30 mM L-lactate as electron donor were used (Shi et al., 2015). For aerobic growth, overnight cultures of *S. oneidensis* strains were inoculated into fresh medium by 200X dilution, shaken at 250 rpm at 30°C, and growth was recorded by measuring optical density at 600 nm (OD_600_).

### In-Frame Mutant Construction and Complementation

In-frame deletion strains were constructed using the *att*-based fusion PCR method as described previously (Jin et al., 2013). In brief, two fragments flanking the target gene were amplified by PCR with primers containing *attB* and the gene specific sequence, and then linked by a second round of PCR. The fused fragments were introduced into plasmid pHGM01 using the Gateway BP clonase II enzyme mix (Invitrogen) according to the manufacturer’s instruction. The resulting vectors were maintained in *E. coli* DAP auxotroph WM3064 and subsequently transferred into relevant *S. oneidensis* strains via conjugation. Integration of the deletion construct into the chromosome was selected by resistance to Gm and confirmed by PCR. Verified transconjugants were grown in NaCl-less LB and plated on LB supplemented with 10% sucrose. Gm-sensitive and sucrose-resistant colonies were screened by PCR for intended deletions. Mutants were verified by sequencing the mutated region.

### Quantitative Proteomic Analysis by LC-MS/MS

For sample preparation, 100 ml of MS medium was inoculated with fresh overnight culture by 200X dilution and incubated at 30°C in a shaker (200 rpm) until entering the stationary phase (∼0.8 of OD_600_). Cultures containing roughly the same number of cells (∼10^10^ cells) were centrifuged at 8000 rpm for 3 min at room temperature and pellets immediately frozen in liquid nitrogen and stored at −80°C. Three independent biological replicates were used for proteomic analysis following procedures: (i) protein extraction; (ii) trypsin digestion; (iii) TMT/iTRAQ labeling; (iv) HPLC fractionation; (v) LC-MS/MS analysis; (vi) database search; and (vii) bioinformatics methods by PTM-Biolabs (Hangzhou, China) using iTRAQ (Isobaric Tag for Relative Absolute Quantitation) and TMT (Tandem Mass Tags) technology. Tandem mass spectra were searched against the NCBI database. False discovery rate (FDR) thresholds for protein, peptide, and modification site were specified at 1%. Minimum peptide length was set at 7. For quantification method, TMT-6-plex was selected. Data were further analyzed as described previously (Yuan et al., 2012). *P*-values were calculated by Mann–Whitney test. *P*-values of less than 0.05 were considered as significant. The fold-change cutoff was set when proteins with quantitative ratio change above 1.5 or below 1/1.5. Fold-changes higher than the cutoff were deemed significant.

### Controlled Gene Expression

Controlled gene expression was used in genetic complementation of mutants and assessment of physiological effects of proteins at varying levels. Genes of interest were generated by PCR, cloned into plasmid pHGEN-Ptac under the control of Isopropyl β-D-1-thiogalactoside (IPTG)-inducible promoter P*tac*, and the resultant vectors were transformed into *E. coli* WM3064 (Meng et al., 2018a). After verification by sequencing, the vectors were transferred into the relevant *S. oneidensis* strains via conjugation. Expression of the cloned genes was controlled by IPTG (Abcam, Shanghai) at varying concentrations.

### Droplet Assays

Droplet assays were employed to evaluate growth inhibition and viability on plates (Jiang et al., 2014). Cells of the exponential phase (∼0.4 of OD_600_, the same throughout the study unless otherwise noted) were collected by centrifugation and adjusted to 10^8^ cfu/ml (colony forming unit), which was set as the undiluted (dilution factor 0). Ten-fold series dilutions were prepared with fresh medium. Five microliters of each dilution was dropped onto LB or MS plates containing required agents. The plates were incubated for 24 h or longer before being read. All experiments were conducted at least three times.

### Disk Diffusion Assays

Disk diffusion assays were done similarly to those done previously (Jiang et al., 2014). One hundred microliters of exponential phase cultures was spread onto an agar plate containing required chemicals. Paper disks (diameter, 8 mm) loaded with chemicals under test were placed on top of the agar. The plates were incubated at 30°C for 24 h prior to analysis.

### Chemical Assays

Through this study, protein concentration was determined with a bicinchoninic acid assay kit with bovine serum albumin (BSA) as a standard according to the manufacturer’s instructions (Pierce Chemical). Where needed, standard curves were made with commercial agents each time.

#### Total Iron

Total iron contents were determined with the established method (Riemer et al., 2004). Cells entering the stationary phase were collected, washed with phosphate buffered saline (PBS), and adjusted to similar densities (∼0.6 of OD_600_). Aliquots of 50 ml were mixed with 5 ml of 50 mM NaOH and sonicated on ice, and centrifuged at 5000 rpm for 10 min. The cell lysates (100 μl) were then mixed with 100 μl 10 mM HCl and 100 μl iron releasing reagent (a freshly mixed solution of equal volumes of 1.4 M HCl and 4.5% [w/v] KMnO_4_) and treated at 60°C for 2 h. After cooling, the iron detection reagents (6.5 mM ferrozine, 6.5 mM neocuproine, 2.5 M ammonium acetate, and 1 M ascorbic acid in water) were added. The absorbance of samples was measured at 550 nm 30 min later. The standard curve was developed using FeCI_3_ up to 300 μM.

#### Free Iron

Quantification of free iron was carried out by electron paramagnetic resonance (EPR) spectroscopy (Keyer and Imlay, 1996). Cultures entering the stationary phase (500 ml) were collected by centrifugation and the cell pellets were resuspended in 0.7% of the original volume of the same medium containing 20 mM desferrioxamine. The sample was incubated at 37°C with shaking for 10 min, centrifuged, washed with cold 20 mM Tris–HCl, pH 7.4, and resuspended in 200 μl of the same buffer containing 10% glycerol. The suspension was scanned with a Bruker EMX plus 9.5/12 spectrophotometer with settings described before (Keyer and Imlay, 1996). Concentrations of intracellular free iron were also assessed by measuring streptonigrin susceptibility with disk diffusion assays.

#### Heme *b* and *c*

Cells entering the stationary phase (∼0.8 of OD_600_) were harvested and then were lysed with lysis buffer (0.25 M Tris/HCl, [pH 7.5], 0.5% Triton-X100). The amount of heme *b* or *c* was measured following the procedure described elsewhere (Berry and Trumpower, 1987).

### Enzyme Assays

Cultures entering the stationary phase were collected by centrifugation, washed twice with PBS, resuspended in 1 ml PBS, and disrupted by sonication. Cell debris and unlysed bacteria were removed via centrifugation. When necessary, enzymes assays were conducted in anaerobic buffers within the anaerobic chamber.

(i) Catalase was assayed by measuring H_2_O_2_ consumption. H_2_O_2_ were added to reactions containing 100 mg total protein immediately after extract preparation to a final concentration of 1 mM, and the reaction mixtures were incubated at 30°C. Aliquots were taken and assayed for remaining oxidant in a time-course manner after the treatment began using the ferrous ion oxidation-xylenol orange (FOX) method (Wolff, 1994).

(ii) Cyt bd oxidase. Activity of cyt bd oxidase was assessed by monitoring nitrite tolerance of relevant strains with droplet assay (Fu et al., 2013).

(iii) All metabolic enzymes were assayed in reactions containing 100 mg total protein immediately after extract preparation. Aconitase (AcnB) was assayed with 30 mM citrate in 50 mM Tris–HCl, pH 7.4 by monitoring the production of *cis*-aconitate (240 nm) (Varghese et al., 2003). Dihydroxyacid dehydratase (IlvD) was assayed with 390 μl pre-incubated solutions containing 50 mM Tris (pH 8.0), 10 mM MgCl_2_, and 10 mM D,L-2,3-dihydroxy-isovalerate by monitoring the production of keto acids (240 nm) (Flint et al., 1993). Peptide deformylase (Def) was assayed with 500 μl of 50 mM HEPES buffer (pH 7.5) containing 25 mM NaCl, 10 mM NAD^+^, 1 unit of formate dehydrogenase, and 1 mM formyl-Met-Ala-Ser (Lazennec and Meinnel, 1997). Threonine dehydrogenase (Tdh) was assayed with 500 μl of 50 mM Tris–HCl buffer (pH 8.4), 1 mM NAD^+^, and 30 mM threonine (Boylan and Dekker, 1981). Ribulose 5-phosphate 3-epimerase (Rpe) was assayed by with a 500 μL reaction mixture containing 50 mM glycylglycine buffer, pH 8.5, 5 mM diethylene triamine pentaacetic acid, 1 unit of α-glycerophosphate dehydrogenase and 10 units of triosephosphate isomerase, 1 mM ribose 5- phosphate, and 0.2 mM NADH (Kiely et al., 1973). Absorbance for Def, Tdh, and Rpe activities were measured at 340 nm. Fumarase (FumB) activity was determined from the appearance of fumarate (250 nm) with a reaction mixture containing 50 mM sodium phosphate (pH 7.4) and 50 mM malate (Massey, 1955).

### Analysis of Gene Expression

For qRT-PCR, cells of the exponential phase were harvested by centrifugation and total RNA was isolated using RNeasy Mini Kit (Qiagen) according to the manufacturer’s instructions. The analysis was carried out with an ABI7300 96-well qRT-PCR system (Applied Biosystems) as described previously (Jiang et al., 2014). The expression of each gene was determined from three replicas in a single real-time qRT-PCR experiment. The Cycle threshold (*C*_*T*_) values for each gene of interest were averaged and normalized against the *C*_*T*_ value of the 16s rRNA gene, whose abundance is relatively constant during the log phase. Relative abundance (RA) of each gene was presented (Jiang et al., 2014).

### SDS-PAGE and Western Blotting Assays

Western blotting analysis was performed for detection of His_6_-tagged proteins as previously described (Dong et al., 2012). Cells entering the stationary phase were harvested by centrifugation, washed with PBS (pH 7.0), resuspended in the same buffer, and sonicated. The cell lysates containing the same amount of proteins were subjected to sodium dodecyl sulfate–polyacrylamide gel electrophoresis (SDS-PAGE, 10%). Proteins were then electrophoretically transferred to polyvinylidene difluoride membranes according to the manufacturer’s instructions (Bio-Rad). The gels were blotted for 2 h at 60 V using a Criterion blotter (Bio-Rad). The blotting membrane was probed with a rabbit polyclonal antibody against His_6_-tag. Goat anti-rabbit IgG-HRP (horseradish peroxidase) (Roche Diagnostics) was used as the secondary antibody (1:5,000) and the signal was detected using a chemiluminescence Western blotting kit (Roche Diagnostics). Images were visualized with a UVP imaging system.

### Other Analyses

Student’s *t-*test was performed for pairwise comparisons. Values were presented as means ± standard error of the mean (SEM).

## Results

### Proteomic Profiling of the *S. oneidensis* Δ*fur* Strain

**FIGURE 1:**
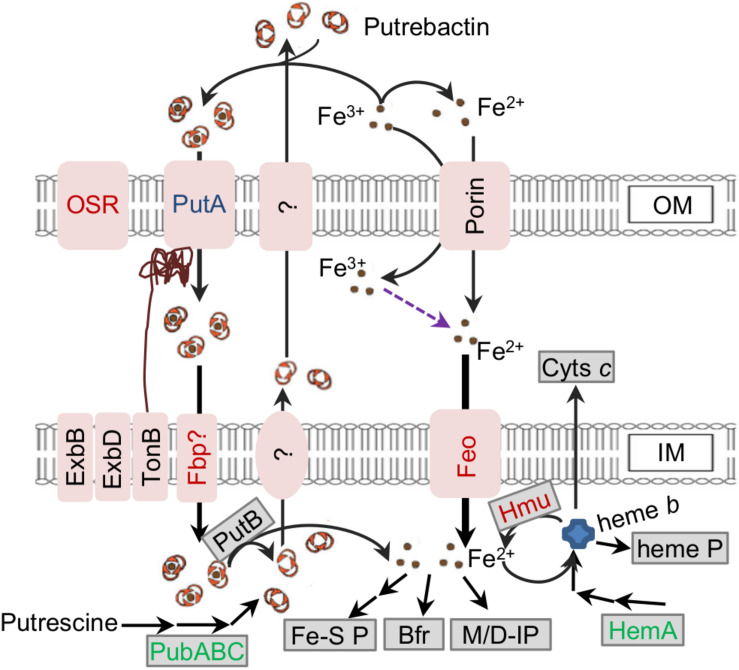
Model for iron homeostasis in *S. oneidensis*. Fe^2+^ (in the environment and from reduction of Fe^3+^) can be imported across IM primarily mediated by Feo as the major route of iron uptake. Additionally, Fe^3+^ can be scavenged by siderophore (putrebactin, produced from putrescine by PubABC and secreted via unknown pathway) to form Fe^3+^-siderophore complexes. The complex enters the periplasm through PutA, and then cross IM via unconfirmed ABC transporter (Fbp?, FbpABC). Fe^3+^ may also be captured by some other bacterial siderophores present in the environment, which enter the cell through other siderophore receptors (OSR). In the cell, Fe^2+^ is released from the complex after the reduction catalyzed by PutB. Fe^2+^ can be stored in Bfr, assembled into Fe-S proteins (Fe-S P) and heme *b* as well as work as a co-factor for mono/di-iron nuclear proteins (M/D IP). Heme *b* can be assembled into heme proteins (heme P) and cyts *c*, and degraded to release Fe^2+^ by HmuRSO (Hmu). Increased, unchanged, and reduced abundance (1.5-fold) in the *fur* mutant based on the proteomic data are in red, blue, and green.

**FIGURE 2:**
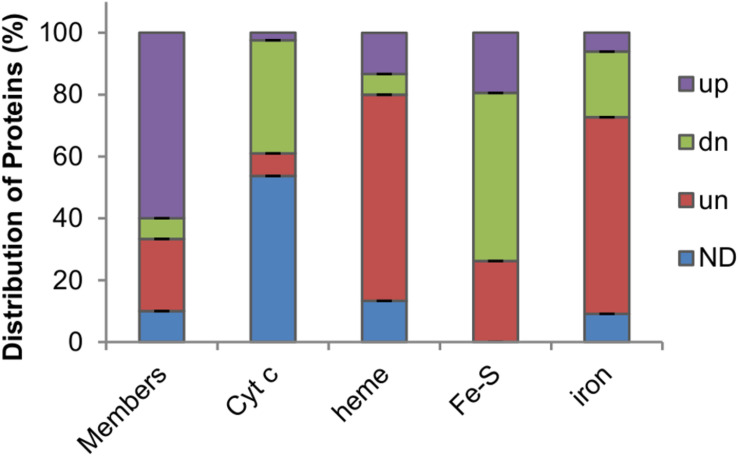
Impacts of Fur loss on levels of Fur regulon members and iron proteins in *S. oneidensis*. Members, proteins whose coding genes carry a Fur-binding motif ranked in top 30. Cyt *c*, cytochrome *c* proteins. Heme, heme-containing proteins other than cyt *c*. Fe-S, proteins carrying any kind of Fe-S clusters. Iron, proteins that bind to and traffic iron or use iron as a cofactor. Up-regulated (up), down-regulated (dn), and unchanged (un) abundance (1.5-fold) in the *fur* mutant based on the proteomic data; ND, no data.

A model illustrating *S. oneidensis* iron homeostasis containing important components was given to facilitate understanding of the relevant biological processes as a whole and to serve as a general guideline for this study (Figure 1). Investigation presented here was initiated by delineating the proteomic differences inflicted by a *fur* deletion. The Δ*fur* and its isogenic parental wild-type strains grown aerobically in defined medium MS to ∼0.8 of OD_600_ (entering the stationary phase) were sampled for proteomics analysis. In total, more than 329 proteins passed the significance threshold (1.5-fold change, *p* < 0.05 of a one-way ANOVA and Tukey’s HSD *post hoc* test). Since the Δ*fur* strain is impaired in growth, abundance of many proteins having roles in general physiology is likely being indirectly affected. To stay focused, only proteins that are predicted Fur regulon members, involved in iron homeostasis, and directly associated with iron cofactor biosynthesis were discussed here (Figure 2 and Supplementary Tables S1, S2).

We first examined differences in the abundance of top-rated (top 30) Fur regulon members, based on the RSAT (Medina-Rivera et al., 2015) weight score of Fur-binding motifs located in promoter regions, between the two strains (Fu et al., 2018). In the Δ*fur* strain, 18 (60%) of them were found to be present in significantly increased quantities whereas only 2 (6.67%) showed decreased abundance (Figure 2 and Supplementary Table S1). This result was in perfect agreement with the proposal that Fur predominantly functions as a transcriptional repressor (Andrews et al., 2003). Clearly, the Fur loss impacted its regulon members that are involved in iron homeostasis most significantly. A large majority of TonB-dependent siderophore receptors (TDSRs) were produced in substantially increased amounts, consistent with the findings that they are induced under iron starvation conditions as well as in the *fur* mutant (Andrews et al., 2003; Fu et al., 2018). These receptors have been proposed to have roles in iron uptake via various siderophores produced by other bacteria in the environment (Liu et al., 2018). In line with this, proteins associated with siderophore-iron uptake, such as FbpABC, were also increasingly produced in the *fur* mutant. On the contrary, Fur-independent TDSR (PutA) for endogenous siderophores, putrebactin, remained rather unaffected by the Fur loss, strongly supporting that the observed differences in protein abundance are largely due to direct regulation of Fur at the transcription level. Expectedly, the Fur loss reduced the quantity of the major iron storage protein Bfr, part of the proposed mechanism that accounts for the iron starvation in the *fur* mutant (Fu et al., 2018).

In the absence of Fur, iron homeostasis is imbalanced: the total iron content decreases but free iron level increases (Fu et al., 2018). This scenario suggests that proteins with iron as a cofactor are likely affected although many of them may not be under the direct control of Fur because of the lack of a Fur-binding motif in their promoter regions. Approximately half of all cyts *c* (19 out of 40) were detected in the proteome (Supplementary Table S1), which is not surprising because numbers of cyts *c* identified in previous proteomics studies are similar or even lower crossing a variety of cultivation conditions (Nissen et al., 2012; Yuan et al., 2012). Among cyts *c* identified, 1 (CcpA), 3, and 15 were up-regulated, unaffected, and down-regulated by the Fur loss, respectively (Figure 2), supporting that Fur is generally required for this group of iron proteins to be produced at physiologically relevant levels. In contrast, most proteins carrying heme molecules via non-covalent bonds, such as catalases and *bd* oxidase (to name a few), were produced at levels comparable to those in the wild-type and Δ*fur* strains (10 out of 15) (Figure 2). As the Fur loss compromises the cyt *c* biosynthesis by lowering heme levels, this observation suggests that these proteins are rather resilient to heme shortage. Intriguingly, all three of the heme-containing proteins with increased abundance in the Δ*fur* strain, regardless of heme-attachment types (covalent or non-covalent), function to decompose hydrogen peroxide (H_2_O_2_), implying that they are concertedly regulated in responsive to oxidative stress.

The largest family of bacterial iron proteins constitute those with Fe-S clusters as cofactors (mostly in 2Fe-2S and 4Fe-4S forms, ∼80 in *E. coli*), most of which play roles in energy-related processes, such as respiration and metabolism (Py and Barras, 2010). According to the genome annotation and the result of BLASTp against the *E. coli* Fe-S proteome, we identified more than 90 Fe-S proteins in *S. oneidensis* (Supplementary Table S2). The majority of these proteins (54.4%) were not significantly affected by the Fur loss in terms of protein abundance, whereas 26.2% and none were found to be present less and more, respectively, in the Δ*fur* strain (Figure 2). Similar results were observed from the last group of iron proteins (33 in total), including mono- and di-nuclear iron enzymes, iron-binding (traffic and storage) proteins, and unspecified iron proteins, all of which do not need an assembly process for their iron molecules. Among them, the majority (64%) was not significantly affected by the Fur loss, whereas those present at decreased and increased levels in the Δ*fur* strain were 7 and 3, respectively (Figure 2). Altogether, the proteomics data suggest that Fur inactivation derepresses of most of its regulon members, imposes critical inhibition on cyt *c* biosynthesis, and modestly and negatively influences proteins with iron cofactors requiring complexation.

### Impacts of the Fur Loss on Activity of Iron Proteins Differ

Iron proteins could be readily divided into two types: Type I iron proteins require an assembly process for their iron molecules to form complex iron cofactors, such as Fe-S clusters and heme, and Type II do not (Solomon et al., 2000; Py and Barras, 2010). Given that the cyt *c* content is significantly reduced upon Fur inactivation (Fu et al., 2018), Type I iron proteins likely suffer from iron shortage. In contrast, as mono- and bi-nuclear iron enzymes exist in the dynamic equilibrium between demetalation and remetallation, the majority of these enzymes are functional at any moment, and thus Type II iron proteins may not be influenced by Fur inactivation (Imlay, 2014).

To test that Fur inactivation differently impacts on activity of these two types of iron enzymes in addition to affecting their quantity, for a comparative analysis we selected two heme proteins, three Fe-S proteins, and three mononuclear iron proteins, whose activities could be specifically evaluated. Two heme proteins were catalase KatB and cyt *bd* oxidase (cyt *bd*) (Fu et al., 2013; Jiang et al., 2014), which differ from cyt *c* in that they carry non-covalent heme and therefore are independent of cyt *c* biosynthesis (Fu et al., 2015). Fe-S proteins included dihydroxy-acid dehydratase (IlvD, 2Fe-2S), aconitase (AcnB, 4Fe-4S), and fumarase (FumB, 4Fe-4S); the former is involved in branched-chain amino acid biosynthesis and the latter two are essential in the TCA cycle (Py and Barras, 2010). Those employing a bound catalytic iron atom (mononuclear) that coordinates substrate included threonine dehydrogenase (Tdh), ribulose 5-phosphate 3-epimerase (Rpe), and peptide deformylase (Def1) (Imlay, 2014). All these iron proteins except for KatB, whose quantity increased 2.49 times, were not significantly affected by Fur inactivation in terms of intracellular abundance (Supplementary Table S2).

**FIGURE 3:**
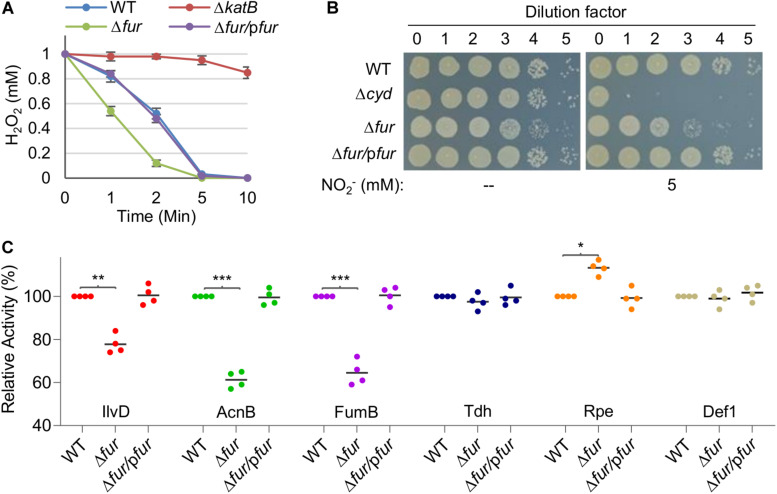
The Fur loss affects activity of iron-proteins differently. **(A)** Activity of heme protein catalase determined by H_2_O_2_ (1 mM) decomposition assay. Cells of indicated strains grown to the mid-exponential phase were collected and adjusted to the same OD values. An aliquot of 200 μl was mixed with H_2_O_2_ and the remaining amounts were determined at indicated times. WT, the wild-type. Δ*fur/*p*fur*, the *fur* null mutant is complemented with a copy of the *fur* gene *in trans*. A mutant lacking catalase KatB, which dictates H_2_O_2_ scavenging capacity of *S. oneidensis*, was used as the negative control. **(B)** Activity of heme protein cyt *bd* oxidase determined by nitrite susceptibility with droplet assays. Cultures at the mid-exponential phase prepared to contain approximately 10^8^ cfu/ml were regarded as the undiluted (dilution factor, 0), which were subjected to 10-fold serial dilution. Five microliters of each dilution was dropped on agar plates with nitrite at indicated concentrations. Results were recorded after 24 h incubation. **(C)** Activity of Fe-S and mono-nuclear proteins determined by respective enzymatic assays. Enzymes included dihydroxy-acid dehydratase (IlvD, 2Fe-2S), aconitase (AcnB, 4Fe-4S), fumarase (FumB, 4Fe-4S), threonine dehydrogenase (Tdh, mono-), ribulose 5-phosphate 3-epimerase (Rpe, mono-), and peptide deformylase (def1, mono-). The activity of each enzyme in the wild-type was set to 100%. Asterisks indicate statistically significant difference of the value compared to that of WT (*P < 0.05; **P < 0.01; ***P < 0.001). Experiments were performed at least three times, and data were presented as means ± SEM or as values representative of similar results.

Catalase assays were performed with cell extracts in order to avoid the interference of electron-dependent peroxidases (Jiang et al., 2014). Results revealed that the Δ*fur* strain had a significantly enhanced capacity of decomposing H_2_O_2_ over the wild-type whereas a *katB* null mutant was largely impotent (Figure 3A). Activity of cyt *bd* was assayed by monitoring susceptibility to nitrite because the enzyme confers *S. oneidensis* cells nitrite tolerance (Fu et al., 2013). The Δ*fur* strain was more sensitive to nitrite than the wild-type, suggesting that the Fur loss impairs activity of cyt *bd* (Figure 3B). Activities of all three Fe-S enzymes under test were significantly lower, by up to 40%, in the Δ*fur* strain than in the wild-type strain (Figure 3C). In contrast, mononuclear enzymes exhibited either comparable or elevated activities in the absence of Fur (Figure 3C). These data suggest that iron proteins carrying complex iron cofactors are subjected to negative regulation when Fur is inactivated.

### The Fur Loss Down-Regulates Heme *b* Levels by Accelerating Heme Degradation

**FIGURE 4:**
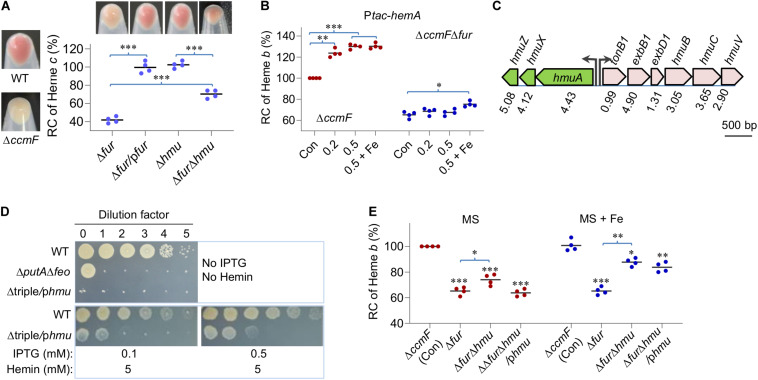
The Fur loss down-regulates heme *b* levels by accelerating heme degradation. **(A)** Heme *c* levels in relevant strains. Cultures of indicated strains entering the stationary phase were pelleted, photographed, and lysed for quantification of heme *c* levels. Δ*hmu*, Δ*hmuAXZ*. RC, relative concentration. The average heme *c* levels in WT and Δ*ccmF* was set to 100% and 0%, respectively. Asterisks indicate statistically significant differences between values linked by bracket (*P < 0.05; **P < 0.01; ***P < 0.001). **(B)** Effects of HemA overproduction on heme *b* levels. Overproduction was driven by the IPTG-inducible P*tac* promoter (P*tac-hemA*) with IPTG at indicated concentrations. Cyt *c*-deficient strains (Δ*ccmF*) were used to avoid the interference of heme *c*, whose heme b level was set to 100%. Con, carrying empty vector. +Fe, supplemented with 0.2 mM FeCl_3_. Asterisks indicate statistically significant difference of the value compared to that of the respective Con. **(C)** The gene organization of heme uptake and utilization in *S. oneidensis*. Proteomic data were shown as the ratio of abundance in Δ*fur* to WT. **(D)** HmuRSO is essential for heme utilization revealed by droplet assays. Δtriple, Δ*putA*Δ*feo*Δ*hmu;* p*hmu*, p*hmuAXZ* driven by P*tac.* **(E)** Effects of *hmuAXZ* deletion on heme *b* levels as assayed as in **(B)**. Experiments were performed at least three times, and data were presented as means ± SEM or as values representative of similar results.

The most evident phenotype resulting from the Fur loss is decolorization of cell pellets, a reflect of substantially compromised cyt *c* biosynthesis (Fu et al., 2018; Figure 4A). Given that the Fur loss down-regulates expression of the *hemA* gene (Figure 1), which encodes the enzyme for the rate-limiting step in heme *b* biosynthesis (Dailey et al., 2017; Fu et al., 2018), we examined the contribution of HemA to the lowered cellular heme *b* levels in the Δ*fur* strain. In an attempt to elevate heme *b* levels by overproducing HemA, expression of the *hemA* gene was driven by IPTG-inducible promoter P*tac* within pHGEN-Ptac (Meng et al., 2018a). To avoid interference of heme *c*, the analysis was carried out in a strain devoid of the *ccmF* gene, which encodes the essential heme lyase for cyt *c* biosynthesis (Kranz et al., 2009; Fu et al., 2015). In the wild-type cells, expression of the *hemA* gene increased heme *b* levels significantly with IPTG at 0.1 mM or higher concentrations (Figure 4B and Supplementary Figure S2). However, the same operation failed to confer the Δ*fur* strain ability to produce more heme *b* with up to 0.5 mM IPTG. Moreover, in the presence of extra iron, the heme *b* levels were only modestly enhanced (Figure 4B). These data suggest that the lowered heme *b* levels in the Δ*fur* strain may not be critically determined by heme *b* biosynthesis.

The proteomic data revealed that the hemin uptake pathway was highly up-regulated upon Fur loss (Figure 4C). The pathway is composed of an outer-membrane receptor (HmuA), an energy transducing system (TonB1-ExbB1-ExbD1) located in the inner membrane, an ATP transporter system (HmuT-HmuU-HmuV), a cytoplasmic hemin binding-shuttling protein (HmuX), and a heme oxygenase (HmuZ) (Richard et al., 2019; Figure 1). Given that there is little exogenous hemin for uptake under experimental conditions, we hypothesized that HmuX and HmuZ produced at highly elevated levels may break down heme *b* molecules that are newly synthesized endogenously, resulting in lowered heme *b* levels.

To test this, we first verified the role of the *hmuAXZ* operon in heme utilization. In *S. oneidensis*, PutA is the TDSR specific for its own siderophores and the Feo complex (composed of FeoA and FeoB polypeptides) is a transport system essential for ferrous iron uptake (Dong et al., 2017; Liu et al., 2018; Wang et al., 2020). As a result, a strain lacking *putA* and *feoAB* genes (Δ*putA*Δ*feo*) could survive and grow only when hemin is supplemented as an iron source. In the presence of a copy of the *hmu* (*hmuAXZ*) operon under the control of IPTG-inducible promoter P*tac*, the chromosomal *hmu* operon was deleted from the Δ*putA*Δ*feo* strain. The resulting strain, Δtriple/p*hmu*, was able to grow in the presence of IPTG but could hardly do so without it on plates supplemented with hemin (Figure 4D), indicating that the *hmu* operon is essential for utilizing heme as an iron source.

We then removed the operon from the wild-type and Δ*fur* strains and determined heme *b* levels of the resulting mutants. Deletion of the *hmu* operon in the wild-type background did not significantly affect cell pellet color (Figure 4A) or heme *b* levels (Figure 4E), suggesting that heme degradation is not activated in the wild-type. In contrast, the impact of the HmuAXZ loss on heme *b* levels in the Δ*fur* strain was significant; cell pellets became notably more reddish (Figure 4A) and there was ∼15% increase in heme *b* levels, reaching to 79% of that of the wild-type (Figure 4E). As expected, addition of iron further improved heme *b* levels in strains lacking the *fur* gene (Figure 4E). These observations were attributable to HmuAXZ because the genetic complementation was successful. Overall, the data indicate that the Fur loss lowers levels of heme *b* by down-regulating its synthesis and up-regulating its degradation simultaneously, and the latter appears to deliver a more profound impact.

### The Fur Loss Activates OxyR

**FIGURE 5:**
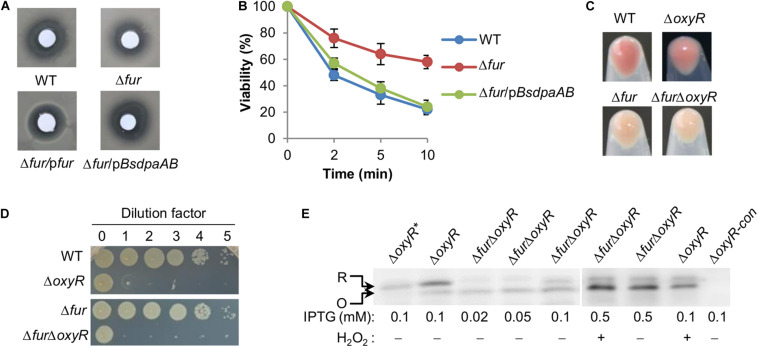
The Fur loss activates OxyR. **(A,B)** Effects of the *fur* mutation on sensitivity of *S. oneidensis* to H_2_O_2_. p*BsdpaAB*, *B. subtilis dpaAB* gene under the control of P*tac* within pHGE-Ptac. Disk diffusion assay **(A)**: paper disks of 6 mm in diameter loaded with 10 μl of 5 M H_2_O_2_ were placed on bacterial lawns (pre-grown for 6 h). Results shown were 24 h after the disks were in place. Survival assay **(B)**: H_2_O_2_ was added to the mid-exponential phase cultures adjusted to 0.4 of OD_600_ to a final concentration of 5 mM and viable cells were counted by plating. **(C,D)** Physiological functions of OxyR and Fur appear non-overlapping as shown by the cell pellet color **(C)** and viability on LB plates **(D)**. **(E)** Immunoblot analysis of OxyR. In the indicated strains, His_6_-tagged protein expression was induced with IPTG. Mid-exponential phase cells either treated with H_2_O_2_ of indicated concentrations for 30 min or not were collected, processed, and analyzed with antibodies against the hexahistidine tag. R, reduced form; O, oxidized form. Experiments were performed at least three times, and representative results are shown. *Represents OxyR^*C203S*^, a mutant that could not be activated. Δ*oxyR*-con, the Δ*oxyR* strain does not express a his6-tagged OxyR as the control.

Based on the data presented thus far, we have drawn a general conclusion that iron proteins with complex iron cofactors are produced less and/or show impaired activity upon Fur inactivation. However, there are exceptions. Heme-containing catalases KatB, KatG-1, and KatG-2 are produced more in the Δ*fur* strain than the wild-type although the latter two do not have detectable physiological activity (Jiang et al., 2014). Moreover, the same occurs to cyt *c* peroxidase CcpA, the only cyt *c* that is up-regulated upon Fur loss. Although *S. oneidensis* has a contracted OxyR regulon, which contains only five members (Jiang et al., 2014; Wan et al., 2018), *katB*, *katG1*, and *ccpA* belong to the OxyR regulon. Moreover, two remaining members, DpsA and AhpCF, were produced at increased levels in the *fur* mutant too. These data imply that the Fur loss activates OxyR-mediated oxidative stress response. To test this, susceptibility of the Δ*fur* strain to H_2_O_2_ was examined. By using disk diffusion assay, we found that the Δ*fur* strain was significantly more resistant to H_2_O_2_ than the wild-type (Figure 5A). Similarly, survival assay revealed that the Fur loss greatly enhanced viability of *S. oneidensis* during H_2_O_2_ killing (Figure 5B).

According to the proteomic data, the Fur loss does not affect intracellular levels of OxyR. However, there is a putative, albeit low-confident, Fur-binding box in the promoter region of the *oxyR* gene based on weight scores obtained from motif screening with RSAT (Fu et al., 2018), implying a possibility of direct regulation by Fur. We thus set out to examine the impact of Fur on transcription of the *oxyR* gene with qRT-PCR. Transcript abundance of the *oxyR* gene was not significantly different in the wild-type and Δ*fur* strains under same conditions, contrasting that of the *bfr* operon used as the positive control (Supplementary Figure S3). Thus, both proteomic and transcriptomic data agree that the possibility of the *oxyR* gene being significantly regulated by Fur in terms of expression is very slim.

Then we examined activation of OxyR by the Fur loss by immunoblot analysis because OxyR proteins in reduced and oxidized forms migrate differently on SDS-PAGE (Wan et al., 2018). For this analysis, we created a Δ*fur*Δ*oxyR* strain from the Δ*oxyR* strain. The double deletion strain displayed phenotypes combining those from both deletions, whitened culture color, and reduced viability on LB plates (Figures 5C,D), implying that their functions are not considerably overlapping. Cells of the Δ*oxyR* and Δ*fur*Δ*oxyR* strains expressing His_6_-tagged OxyR (driven by P*tac*), which is fully functional (Wan et al., 2018), were grown to the mid-exponential phase (∼0.4 of OD_600_) and collected for immunoblot analysis. As shown in Figure 5E, both reduced and oxidized OxyR proteins was found in *fur*^+^ (Δ*oxyR*) cells grown under normal conditions with the reduced form dominating, while negative control OxyR^C203S^, an OxyR mutant that could not form disulfide bond, was present in the reduced form only. In H_2_O_2_-challenged cells, the majority of OxyR proteins became oxidized. In the *fur*^–^ (Δ*fur*Δ*oxyR*) background, either treated with H_2_O_2_ or not, the ratio of OxyR proteins in the oxidized form to in the reduced form increased significantly (Figure 5E). These data support that the Fur loss results in activation of OxyR without significantly affecting its production.

### The Feo System Is Partially Responsible for Elevated Free Iron Levels in *S. oneidensis fur* Mutants

**FIGURE 6:**
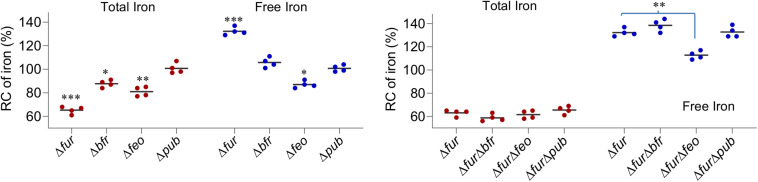
The Feo system has a role in elevated free iron levels caused by the Fur loss. Effects of indicated deletions on cellular iron levels. Cultures of indicated strains entering the stationary phase were collected and their total iron and free iron contents were determined. The averaged levels of WT were set to 100%. Asterisks indicate statistically significant differences of the value compared to that of the WT and between values linked by bracket (*P < 0.05; **P < 0.01; ***P < 0.001). Experiments were performed at least three times, and data were presented as means ± SEM.

Previous analyses have shown that concentrations of the total iron and free iron in the *S. oneidensis* Δ*fur* strain decrease by ∼25% and increase by ∼35%, respectively, compared to those in the wild-type (Fu et al., 2018). To unravel the underpinning mechanism, we looked into iron storage proteins because their *E. coli* functional counterparts are responsible for the same phenomenon (Andrews et al., 2003). In *S. oneidensis*, bacterioferritin (Bfr) is the predominant Fur-dependent iron storage protein complex whereas neither ferritin (Ftn) nor DpsA plays an important role (Fu et al., 2018; Figure 1). The removal of Bfr from the wild-type significantly reduced the levels of total iron but had little effect on free iron (Figure 6). In the absence of Fur, however, the contribution of Bfr became diminished (Figure 6), an expected result because of its dependence on Fur for production (Fu et al., 2018). These data suggest that, upon Fur inactivation, *S. oneidensis* cells restrain iron levels not likely by limiting iron storage.

We then turned to iron uptake. In *S. oneidensis*, iron uptake is predominantly mediated by the ferrous iron uptake system (Feo) (Liu et al., 2018; Figure 1). According to the proteomic data, in the *fur* mutant the Feo system was modestly up-regulated, FeoA and FeoB, 1.27- and 1.89-fold, respectively, suggesting that this system may not be functionally compromised by the Fur loss. The contributions of the system in iron uptake in both the wild-type and Δ*fur* strains were then assessed. In the *fur*^+^ background, the loss of the Feo system modestly reduced both the total and free iron levels (Figure 6). In the Δ*fur*Δ*feo* strain, the free iron level decreased substantially but the total iron level remained comparable to that of the *fur* mutant (Figure 6). Reduced free iron levels in the Δ*feo* and Δ*fur*Δ*feo* strains were verified by enhanced resistance to streptonigrin (SNG) (Supplementary Figure S4), a redox cycling antibiotic whose antibacterial activity correlates with the levels of intracellular free iron (Wilson et al., 1998). Clearly, the Feo system is critical for elevated free iron levels caused by the Fur loss.

Next to the Feo system is the siderophore-dependent system for iron uptake in *S. oneidensis* (Dong et al., 2017). This system is composed of enzymes for biosynthesis of siderophores (PubABC) and proteins for their transport (PutA) and reduction (PutB, reductase), all of which are functionally essential (Liu et al., 2018; Wang et al., 2020; Figure 1). Among them, enzymes for siderophore synthesis (PubABC, 0.96, 0.36, and 0.48-fold, respectively) were less abundant than in the wild-type, as revealed by the proteomic analysis (Supplementary Table S1). The reduction in the quantity of the PubABC enzymes concurs with the previous finding that the *fur* mutant secretes siderophores at significantly lowered levels (Fu et al., 2018). Consistently, PutA was not affected by the *fur* mutation, although most of other TDSRs were highly up-regulated (Supplementary Table S1 and Figure 1). To test if the lowered production of siderophore molecules plays a role in altered iron levels caused by Fur inactivation, we monitored iron levels in the Δ*pub* and Δ*fur*Δ*pub* strains. Results demonstrated that the siderophore-deficient mutation had no detectable influence on either total or free iron levels in either the wild-type or *fur* mutant (Figure 6). Thus, the possibility that the siderophore-dependent system is a major contributing factor in *S. oneidensis* iron homeostasis was eliminated.

### The Free Iron Content Determines the Physiological Impacts of the Fur Loss

No matter how Fur inactivation alters total iron contents in all reported cases, it elevates intracellular free iron levels. This prompts us to speculate that the elevated free iron content is a dictating factor for the observed phenotypes resulting from the Fur loss. To test this idea, effects of iron chelators, to which the cell membrane is permeable and impermeable, on the *fur* mutant were compared. We reasoned that the influence of desferrioxamine (DFO), a commercially available siderophore that cannot be imported into *S. oneidensis* cells and thus functions to abruptly terminate iron uptake (Liu et al., 2018), should be different from that of cell-permeant 2-2-dipyridyl, which is potentially able to lower free iron in the cytoplasm.

**FIGURE 7:**
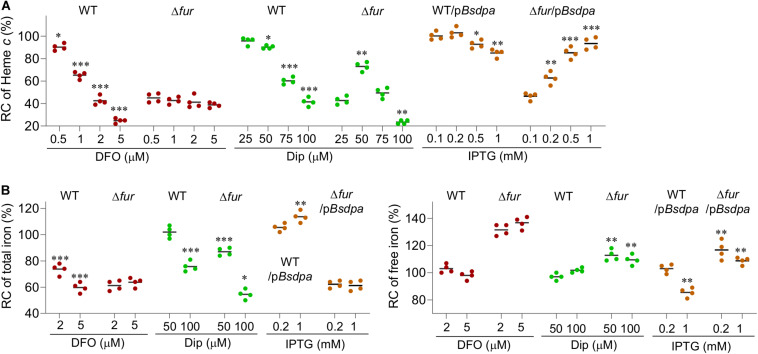
The free iron content determines the physiological impacts of the Fur loss. **(A)** Suppressing effects of iron chelators on the *fur* deletion based on cyt *c* biosynthesis. Dip, 2,2-dipyridyl. p*BsdpaAB*, *B. subtilis dpaAB* gene under the control of P*tac* within pHGE-Ptac. **(B)** Relative concentrations (RC) of total and free iron in WT and Δ*fur* strains. Cultures of indicated strains entering the stationary phase were collected and their total iron and free iron contents were determined. The averaged levels of WT were set to 100%. Con, no chelator; DFO-2 and -5, DFO at 2 and 5 μM; Dip-50 and -100, dipyridyl at 50 and 100 μM; IPTG-0.2 and -1, p*BsdpaAB* with IPTG 0.2 and 1 mM. Asterisks indicate statistically significant differences of the value compared to that of the WT and between values linked by bracket (*P < 0.05; **P < 0.01; ***P < 0.001). Experiments were performed at least three times, and data were presented as means ± SEM or by representative results.

At 2 and 5 μM, DFO decreased cyt *c* biosynthesis (whitened cell pellets, Supplementary Figure S5) and iron content of the wild-type significantly while the effect was not evident at lower concentrations (Figures 7A,B). Despite this, even 5 μM DFO barely altered free iron concentrations evidenced by similar resistance to SNG (Supplementary Figure S4). In the Δ*fur* strain, cyt *c* biosynthesis (the color of cell pellets, Fig. S5) was not notably affected by DFO at all test concentrations (Figure 7A). In line with this, neither total nor free iron content in the *fur* mutant was responsive to DFO treatment (Figure 7B and Supplementary Figure S4). When 2,2-dipyridyl was used, the wild-type strains responded similarly as DFO in terms of cyt *c* biosynthesis (cell pellet color, Supplementary Figure S5) and total iron content (Figures 7A,B). However, free iron content decreased with 2,2-dipyridyl at 50 μM and above (Figure 7B and Supplementary Figure S3). The Δ*fur* strain displayed a different scenario in responding to 2,2-dipyridyl. The cyt *c* biosynthesis increased (the cell pellet color becoming increasingly reddish, Supplementary Figure S5) with concentrations closing to 50 μM, returned to the original with 75 μM, and became even paler with 100 μM. Although 50 μM 2,2-dipyridyl did not significantly lower the total iron content, it reduced the free iron levels substantially (Figure 7B), conferring cells elevated resistance to SNG (Supplementary Figure S4).

Although the different effects of 2,2-dipyridyl on the wild-type and Δ*fur* strains strongly support that the free iron content is important for the physiological impacts of the Fur loss, additional evidence is needed because the chelator reduces extracellular iron levels simultaneously. To circumvent this, in *S. oneidensis* we engineered production of dipicolinate, an iron chelator that could not cross cell membranes because it is a charged molecule (Maringanti and Imlay, 1999). *Bacillus subtilis* dipicolinate synthetase (encoded by *BsdpaAB*) is able to carry out one-step conversion of dipicolinate from dihydrodipicolinate, an intermediate in the pathway of diaminopimelate and lysine biosynthesis (Hutton et al., 2007; Supplementary Figure S6). Overproduction of *Bs*DpaAB in *E. coli* could substantially reduce free iron levels (Maringanti and Imlay, 1999). We placed the *BsdpaAB* under the control of P*tac* within pHGE-Ptac and introduced the resulting vector into relevant *S. oneidensis* strains. The cyt *c* biosynthesis (cell pellet color, Supplementary Figure S5) of the wild-type was not affected by this vector with up to 1 mM IPTG (saturated induction) (Figure 7A). In contrast, this vector significantly suppressed the cyt *c* biosynthesis defect (the cell-pellet color, Supplementary Figure S5) of the Δ*fur* strain in the presence of IPTG at 0.2 mM or higher (Figure 7A), exactly like that observed from 50 μM 2,2-dipyridyl. However, cyt *c* biosynthesis additionally increased slightly (the cell pellet color remained reddish, Supplementary Figure S5) with further increased *Bs*DpaAB production, contrasting the effect of 2,2-dipyridyl at higher concentrations. Quantification of iron contents demonstrated that *Bs*DpaAB produced with 0.2 mM IPTG increased the total iron content but did not alter the free iron levels in the wild-type (Figure 7B and Supplementary Figures S4, S5). In the Δ*fur* strain, we found that the total iron content was barely affected but the free iron levels decreased significantly when *Bs*DpaAB was produced to sufficient amounts. In addition, we found that manipulated production of *Bs*DpaAB was also able to suppress the effects of Fur loss on oxidative stress response (Figures 5A,B). These data, all together, conclude that excessive free iron is largely accountable for the physiological impacts of the Fur loss.

## Discussion

In many bacteria, Fur proteins are now regarded as global transcriptional regulators, controlling a large number of genes participating in diverse biological processes, such as redox regulation, energy metabolism, defenses against oxidative and nitrosative stresses, nucleic acid biosynthesis, cell morphology and motility, and many more (Jittawuttipoka et al., 2010; Yu and Genco, 2012; Becerra et al., 2014; Fillat, 2014; González et al., 2014; Seo et al., 2014; Pasqua et al., 2017). Fur inactivation imposes upon bacterial cells an inability to maintain normal iron homeostasis, resulting in altered iron contents that triggers an array of physiological abnormalities. In metal-reducing bacteria, Fur regulation appears particularly important as iron is the essential cofactor for electron transport chain components and terminal reductases, especially cyts *c* (Senko and Stolz, 2001; Thompson et al., 2002; Fredrickson et al., 2008).

The primary objective of the present study was to clarify how pleiotropic regulator Fur regulates activity of iron-containing proteins of *S. oneidensis* and to define the essence of the Fur regulation. In *S. oneidensis*, while early transcriptomics studies validated Fur as the predominant regulator for iron homeostasis (Wan et al., 2004; Yang et al., 2008), recent investigations expanded its impacts beyond iron-related processes, including induction of λSo prophage and production of outer-membrane porins (Binnenkade et al., 2014; Gao et al., 2015). Here, we have examined influences of the Fur loss on an array of iron-containing proteins, an idea stemming from compromised overall cyt *c* biosynthesis (Fu et al., 2018).

In *S. oneidensis*, heme *b* is an essential molecule because respiration of all EAs known to date, including oxygen and non-oxygen ones, is absolutely dependent on heme proteins (Fu et al., 2015). Fur is involved in regulation of heme *b* biosynthesis, a finding that is expected because the intracellular iron content is crucial for biosynthesis of heme *b* (Dong et al., 2017). It has been previously proposed that Fur influences heme *b* biosynthesis by affecting transcription of *hemA*, whose product catalyzes the rate-limiting step of heme *b* biosynthesis (Dailey et al., 2017; Liu et al., 2018). However, here, we reveal an additional and more important mechanism accounting for the heavily reduced heme *b* content in the *fur* mutant. Genes for heme (hemin) uptake and utilization proteins are conserved members of the Fur regulon in many bacteria, including *S. oneidensis* (Fu et al., 2018; Richard et al., 2019). Derepression of these genes by Fur inactivation under iron starvation conditions is a strategy for bacterial cells to obtain heme as an alternative iron source. Clearly, *S. oneidensis* adopts the same mechanism. Under experimental conditions, heme molecules in the extracellular milieu are too low to meet the demand of the heme utilization reductase and oxygenase, which are overproduced along with the heme import system in cells of the *fur* mutant. Instead, heme molecules generated endogenously appear to be victims of these utilization proteins (Richard et al., 2019), reducing intracellular heme levels. This mechanism also reasonably explains the finding that the *fur* mutant carries more severe defects in cyt *c* biosynthesis than a strain lacking Crp, the most important global regulator for respiration (Saffarini et al., 2003; Yin et al., 2016; Fu et al., 2018). In *S. oneidensis*, the *hemA* gene is subject to positive regulation of Crp too and the Crp loss compromises *hemA* expression at least comparably, if not more severely, to the Fur inactivation. However, as Crp does not control heme uptake and utilization systems, heme levels in the *crp* mutant exceed those in the *fur* mutant (Yin et al., 2016; Fu et al., 2018).

Despite reduced production and enhanced decomposition, heme is still maintained at levels sufficiently high to ensure essential or crucial heme proteins to function in the *fur* mutant. It is common that multiple terminal oxidases are encoded in a bacterial genome, but one suffices to respire oxygen and therefore to support growth under aerobic conditions (Zhou et al., 2013). In *S. oneidensis*, the primary oxidase for oxygen respiration is cyt *c* oxidase *cbb*_3_ while cyt *bd* oxidase functions as an auxiliary enzyme to reduce oxygen but dictates nitrite tolerance (Fu et al., 2013; Zhou et al., 2013). In the absence of Fur, the primary oxidase, cyt *c* oxidase *cbb*_3_, is substantially impaired; to survive and to grow, cells have to rely on cyt *bd* oxidase for oxygen respiration (Fu et al., 2018). The proteomic data show that the quantity of cyt *bd* is barely affected by the Fur loss. This concurs with the previous observations that there is no compensating production for cyt *bd* when cyt *cbb*_3_ is depleted (Yin et al., 2016; Zhang et al., 2020). Nonetheless, activity of cyt *bd* is significantly impaired upon Fur inactivation, evidenced by increased susceptibility to nitrite (Fu et al., 2013; Meng et al., 2018b; Zhang et al., 2020). We therefore suggest that Fur inactivation results in heme *b* shortage, which retards the assembling process for cyt *bd* to be a functional enzyme. By the same token, the Fur loss causes reduced activity of F-S enzymes tested in this study as biosynthesis of iron-sulfur clusters is also a complex process involving a consortium of highly conserved proteins (Frazzon et al., 2002). On the contrary, Fur hardly shows noticeable impact on production and activity of proteins that use iron atom as cofactors directly, such as mononuclear proteins.

In bacteria, it is well established that the control of iron homeostasis and responses to oxidative stress are coordinated (Cornelis et al., 2011). *E. coli fur* mutants were found to be hypersensitive to H_2_O_2_ more than 2 decades ago (Touati et al., 1995), and since then, similar phenomena have been observed in most, if not all, of the bacteria in which the subject was studied (Cornelis et al., 2011). The underpinning mechanism is that Fur inactivation leads to unrestrained iron uptake and diminished iron consumption due to reduced production of iron proteins, resulting in elevated iron contents, free iron in particular, which eventually promotes hydroxyl radical generation via Fenton chemistry. However, this is not the case in *S. oneidensis*. The OxyR regulon of *S. oneidensis* is rather small, containing only 5 members (Jiang et al., 2014; Wan et al., 2018). Four of them are iron-containing proteins, catalases KatB and KatG1, cyt *c* peroxidase CcpA, and iron-storage protein DpsA, all of which display increased expression in the *fur* mutant, in contrast to most of their kind. In addition, the only non-iron member of the OxyR regulon, AhpCF (NADH peroxidase), is also present in increased quantity. We show here that the Fur loss modulates the redox status rather than the quantity of OxyR proteins, which exist in both reduced and oxidized forms at the same time (Wan et al., 2018). By reducing the ratio of the reduced to the oxidized, the Fur depletion results in overall activation of the regulator. Thus, although the free iron level becomes elevated upon Fur inactivation in *S. oneidensis*, cells manage to trigger prompt responses to oxidative stress for protection. We speculate that evolution has honed *Shewanella* for their strategy to copy with excessive iron because they live in redox-stratified niches and contain unusually high concentrations of iron comparing to model bacteria, such as *E. coli* (Daly et al., 2004; Jiang et al., 2014).

A common consequence of the Fur loss is iron overload, a scenario that is in line with the repressing role of Fur for iron acquisition (Vajrala et al., 2011; Latorre et al., 2018). Although a large portion of these studies do not distinguish between free iron and iron bound to proteins, it is reasonable to assume that the free iron content increases, as revealed in a few (Jittawuttipoka et al., 2010; Latorre et al., 2018). An increase in the free iron content upon Fur inactivation is also observed in some other bacteria, such as *E. coli* and *S. oneidensis*, despite a decrease in the total iron content (Keyer and Imlay, 1996; Abdul-Tehrani et al., 1999). To date, exceptions to elevated free iron content resulting from the Fur loss have been exclusively observed in α-proteobacteria, such as *Bradyrhizobium japonicum*, *Rhizobium leguminosarum*, *Agrobacterium tumefaciens*, and so forth (O’Brian, 2015). In these bacteria, Fur-like proteins have either been initially found to physiologically function in response to other metals or senses iron indirectly (Qi and O’Brian, 2002; O’Brian, 2015). Thus, it seems that regulation of response to iron availability in α-proteobacteria evolves differently from other bacteria employing Fur as the master iron-responsive regulator, in which Fur inactivation generally leads to increased free iron content.

In this study, our data suggest that the increased free iron content upon Fur loss is the key to trigger the physiological abnormalities. The first piece of evidence comes from the observation that membrane-permeable chelator dipyridyl but not impermeable DFO, when present at proper levels, could restore cyt *c* biosynthesis in the *fur* mutant. The successful suppression of the cyt *c* biosynthesis defect by dipicolinate, a chelator that functions only intracellularly, provides more rigid support. Although the connections between the elevated free iron content and Fur inactivation remain to be uncovered, we speculate that the presence of excessive free iron would signal and prevent more iron uptake despite an up-regulated iron uptake system in the *fur* mutant. Despite this, given that the cellular storage capacity is heavily discounted, the *fur* mutant would maintain a high level of free iron content. It should be noted that the cellular storage proteins, even forcibly produced to considerable quantity, are not effective as chelators tested here (Fu et al., 2018). We do not yet understand the mechanism behind this. One may imagine that the free iron pool is restricted by both iron storage proteins and small molecule chelators in the wild-type cells and production of both is compromised by Fur activation. We are working to test this notion.

## Data Availability Statement

The mass spectrometry proteomics data have been deposited to the ProteomeXchange Consortium (http://proteomecentral.proteomexchange.org) via the iProX partner repository with the dataset identifier PXD021732.

## Author Contributions

LL, XF, WW, YC, and ZC conducted and performed the experiments. LL and XF contributed to data discussion and analysis. HG designed and supervised the study, and wrote the manuscript with LL. All authors contributed to the article and approved the submitted version.

## Conflict of Interest

The authors declare that the research was conducted in the absence of any commercial or financial relationships that could be construed as a potential conflict of interest.
