# Supplementary material for: Free Rather Than Total Iron Content Is Critically Linked to the Fur Physiology in Shewanella oneidensis
Source: Front Microbiol. 2020 Nov 26;11:593246. doi: 10.3389/fmicb.2020.593246 (PMC7732582; doi:10.3389/fmicb.2020.593246)
Supplement: Supplementary file 1 [file Data_Sheet_1.PDF]

```
Pa-Fur      -MVENSELRKAGLKVTLPRVKILQMLDSAEQRHMSAEDVYKALMEAGEDVGLATVYRVLT    59
Ec-Fur      MTDNNTALKKAGLKVTLPRLKILEVLQEPDNHHVSAEDLYKRLIDMGEEIGLATVYRVLN    60
St-Fur      MTDNNTALKKAGLKVTLPRLKILEVLQEPDNHHVSAEDLYKRLIDMGEEIGLATVYRVLN    60
So-Fur      MTDGNQALKKAGLKITLPRVKILELMQGPENQHISAEDLYKKLLDLGEEIGLATVYRVLN    60
Vc-Fur      MSDNNQALKDAGLKVTLPRLKILEVLQQPECQHISAEELYKKLIDLSEEIGLATVYRVLN    60
                *  *:.****:****:***::::  : :*:***::** *:: .*::*********.

Pa-Fur      QFEAAGLVVRHNFDGGHAVFELADSGHHDHMVCVDTGEVIEFMDAEIEKRQKEIVRERGF    119
Ec-Fur      QFDDAGIVTRHNFEGGKSVFELTQQHHHDHLICLDCGKVIEFSDDSIEARQREIAAKHGI    120
St-Fur      QFDDAGIVTRHNFEGGKSVFELTQQHHHDHLICLDCGKVIEFSDDSIEARQREIAAKHGI    120
So-Fur      QFDDAGIVSRHHFESGKAVFELSTQHHHDHLVCLSCGKVIEFSDEVIERRQDEIASKYNI    120
Vc-Fur      QFDDAGIVTRHHFEGGKSVFELSTQHHHDHLVCLDCGEVIEFSDDVIEQRQKEIAAKYNV    120
            **: **:* **:*:.*::****: . ****::*:. *:**** *  ** ** **. : ..

Pa-Fur      ELVDHNLVLYVRKKK---------------    134
Ec-Fur      RLTNHSLYLYGHCAEGDCREDEHAHEGK--    148
St-Fur      RLTNHSLYLYGHCAEGDCREDEHAHDDATK    150
So-Fur      KLTNHSLYLYGHCTNDNCEHNDE-------    143
Vc-Fur      QLANHSLYLYGKCGSDGSCKDNPNAHKPKK    150
            .*.:*.* ** :  .
```

Fig. S1. Amino acid sequence alignment of Fur proteins from *Pseudomonas aeruginosa* (*Pa*), *E. coli* (*Ec*), *S. oneidensis* (*So*), *Salmonella enterica* serovar Typhimurium (*St*), and *Vibrio cholerae* (Vc). Sequence identities among these proteins are above 70% with BLASTp E-value smaller than $1e^{-70}$. More importantly, the Fe binding sites are perfectly conserved: in the case of *E. coli*, the Fe atom is secured by 3 His (His33, His88, and His90) and 2 Glu (Glu81 and Glu101), which are conserved (highlighted) in all Fur proteins.

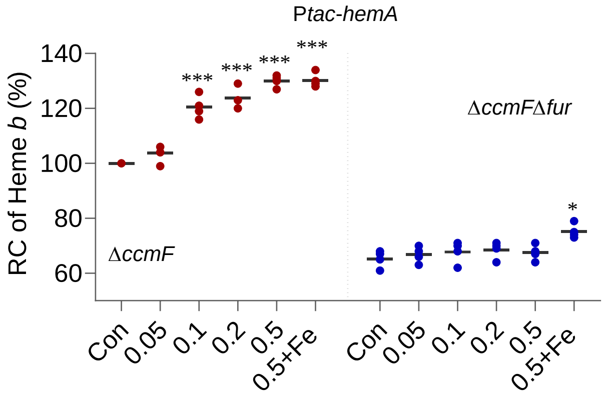


**FIG S2 Effects of HemA overproduction on heme *b* levels.** Overproduction was driven by the IPTG-inducible P*tac* promoter (P*tac-hemA*) with IPTG at indicated concentrations. Cyt *c*-deficient strains (Δ*ccmF*) were used to avoid the interference of heme *c*, whose heme b level was set to 100%. Con, carrying empty vector. + Fe, supplemented with 0.2 mM $FeCl_3$. Asterisks indicate statistically significant difference of the value compared to that of the respective Con. Asterisks indicate statistically significant differences between values linked by bracket (n = 4, *, $P < 0.05$; **, $P < 0.01$; ***, $P < 0.001$).

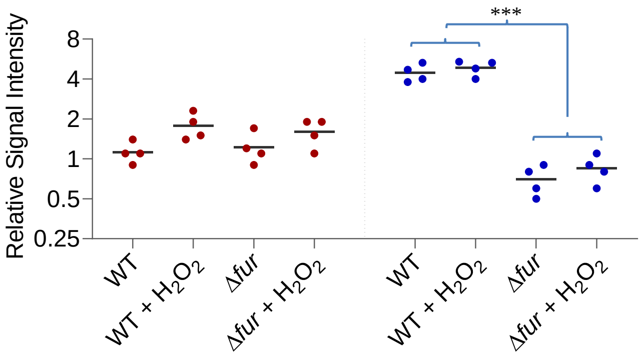


**FIG S3 Fur does not affect transcription of the *oxyR* gene in *S. oneidensis*.** Shown were data from qRT-PCR analysis of RNA extracted from mid-log-phase growing cells before and after $H_2O_2$ treatment (0.2 mM for 2 min). All data were normalized to expression levels of the *arcA* gene, which were constant during the exponential growth phase. The *bfr* operon, a member of the Fur regulon in *S. oneidensis*, was used as the positive control. Asterisks indicate statistically significant differences between values linked by bracket (n = 4, *, $P < 0.05$; **, $P < 0.01$; ***, $P <$ 0.001).

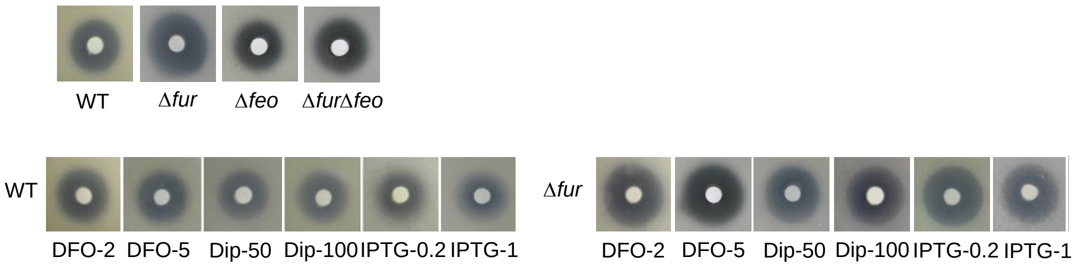


**FIG S4 Free iron levels reflected by** susceptibility to streptonigrin (SNG) by the diffusion method. Ten microliters of 2 mg/ml SNG was added to filter paper discs (8 mm) on cultures of indicated strains. Plates were incubated at 30° C for 24 h. IPTG, for production of p*BsdpaAB*, *B. subtilis dpaAB* gene under the control of P*tac* within pHGE-Ptac (refer to Fig. 7 for details). Experiments were performed at least three times, and representative results were shown.

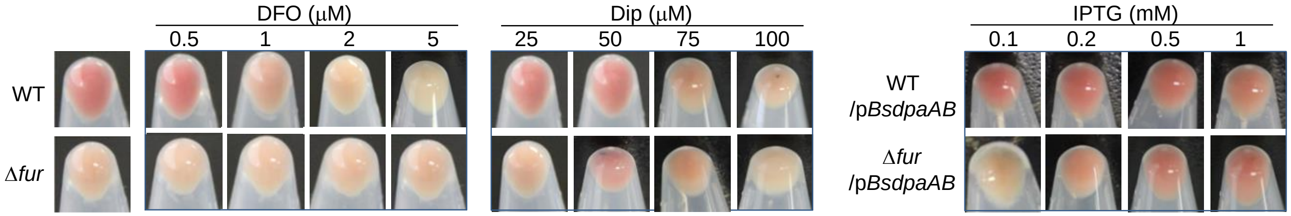


**FIG S5 Suppressing effects of iron chelators on the *fur* deletion based on cyt *c* biosynthesis.** Dip, 2,2-dipyridyl. p*BsdpaAB*, *B. subtilis dpaAB* gene under the control of P*tac* within pHGE-Ptac. Experiments were performed at least three times, and representative results were shown.

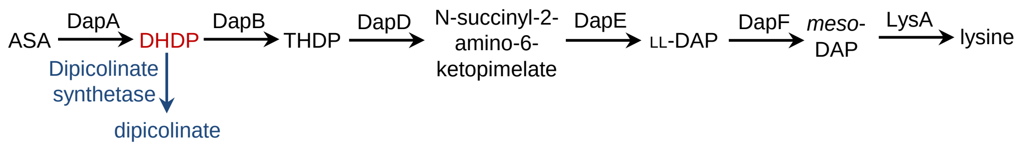


**FIG S6 Pathway of diaminopimelate and lysine biosynthesis.** The conversion of dihydrodipicolinate (DHDP) to dipicolinate by *B. subtilis* dipicolinate synthase was shown in blue. ASA, aspartate semialdehyde; THDP, tetrahydrodipicolinate, LL-DAP, L,L-diaminopimelate

TABLE S1 Abundance difference of top-rated of Fur regulon members

| Gene | Locus | Annotation | Ratio of Δ*fur* /WT | *p* value | Motif | Weight |
|---|---|---|---|---|---|---|
| SO_2366 | SO_2366 | two component system response regulator with DUF3369 and HD domains | ND | | ATTAATGATAACGATTATC | 14.1 |
| irgA | SO_4523 | iron-responsive TonB-dependent enterobactin receptor IrgA | 4.971 | 0.116 | GGTATTGAAAATTATTATC | 13.2 |
| SO_1482 | SO_1482 | TonB-dependent siderophore receptor | 3.5145 | 0.159 | GAGGATGGAAATCATTATC | 12.6 |
| fbpA | SO_0744 | ABC-type Fe3+ uptake system substrate-binding component FbpA | 2.039 | 0.007 | GAAACTGATAACTATTATC | 12.6 |
| SO_3344 | SO_3343 | predicted lipoprotein | ND | | TTAAATGATAATGATTATC | 12.3 |
| SO_1188 | SO_1188 | inner membrane protein with PepSY TM helix | 3.416 | 0.040 | GTAAATGATATTTATTATC | 12 |
| SO_4516 | SO_4516 | TonB-dependent siderophore receptor | 4.058 | 0.058 | GTAAATGATATTGGTTATC | 11.8 |
| SO_3914 | SO_3914 | TonB-dependent siderophore receptor | 4.557 | 0.120 | GCAAATGATAATAATTATT | 11.4 |
| brf2 | SO_1111 | bacterioferritin subunit 2 Bfr2 | 0.578 | 0.270 | GATAATGAGAATGCTTTTA | 11.4 |
| SO_1998 | SO_1998 | protein of unknown function DUF3389 | ND | | CAGTGTGATAATGATTATT | 11.2 |
| dmsA | SO_1429 | extracellular dimethyl sulfoxide/manganese oxide reductase molybdopterin-binding subunit DmsA | 0.242 | 0.062 | GTTTTTGAGAATGATTTTC | 10.9 |
| SO_4743 | SO_4743 | TonB-dependent siderophore receptor | 3.176 | 0.038 | TTTAATGAGAATTGTTATC | 10.8 |
| comEA | SO_1813 | DNA competence protein ComEA | 0.8965 | -144 | GATATTAAGAACCGTTATT | 10.8 |
| exbB | SO_3671 | TonB1 energy transduction system for heme uptake inner membrane component ExbB | 4.898 | 0.072 | GCAAATGATAATGATTTCT | 10.3 |
| hmuA | SO_3669 | TonB-dependent heme/hemoglobin receptor HmuA | 4.347 | 0.129 | GCAAATGATAATGATTTCT | 10.3 |
| cpdB | SO_3565 | bifunctional 2',3'-cyclic-nucleotide 2'-phosphodiesterase/3`-nucleotidase CpdB | 0.8435 | -286 | GTTGATGATAATTAATATT | 10.1 |
| SO_0798 | SO_0798 | TonB-dependent receptor | 3.726 | 0.069 | GTAAATGAGAATTGTTATT | 10 |
| SO_1755 | SO_1755 | phosphoglucomutase/phosphomannomutase family protein | 2.119 | 0.013 | GTAATTGAAAATGATTTTC | 10 |
| SO_1966 | SO_1966 | protein of unknown function DUF124 | 1.006 | -119 | GATAATGATAAAAATAATC | 9.8 |
| feoB | SO_1784 | ferrous iron transport protein B FeoB | 1.893 | 0.067 | GGTAACGATAATAGTTTTC | 9.7 |
| SO_2426 | SO_2426 | two component signal transduction system response regulator | 4.320 | 0.004 | ATAAATGATATTGATTCTC | 9.6 |
| SO_2841 | SO_2841 | hypothetical protein | 2.336 | -56 | CTAAATGAAAACTATTATC | 9.6 |
| SO_1580 | SO_1580 | TonB-dependent haem/haemoglobin receptor | 4.167 | 0.114 | AAAGATGAGAATAATTCTC | 9.5 |
| SO_1579 | SO_1579 | protein of unknown function DUF2461 | 1.176 | -157 | AAAGATGAGAATAATTCTC | 9.5 |
| SO_1380 | SO_1380 | protein of unknown function DUF2913 | 1.0295 | -112 | GTTGGTAAGAATCGTTATC | 9.5 |
| SO_4422 | SO_4422 | TonB-dependent ferric achromobactin receptor | 1.663 | 0.010 | GCAAATGGTAACGATTTTT | 9.4 |
| ftn | SO_0139 | ferritin Ftn | 1.605 | 0.066 | GTTGATAAAAATGATTTTC | 9.4 |
| SO_2247 | SO_2247 | putative lipoprotein | 1.0295 | -119 | GTCGATGATAGCGATTATT | 9.4 |
| SO_3549 | SO_3549 | hemerythrin | 0.8665 | -177 | GATATTGATTAGTCTTATT | 9.4 |
| SO_3407 | SO_3407 | putative siderophore transporter component 2 | 4.195 | 0.014 | GCAAATGATATTGTTTATC | 9.3 |

**TABLE S2 Abundance difference of iron-containing proteins**

| Locus | Gene name | Protein function | Iron cofactor | fur/WT Ratio | fur/WT CV |
|---|---|---|---|---|---|
| SO_0045 | nsrR | nitrite-sensitive transcriptional repressor NsrR | 2Fe-2S | 0.91 | 0.001 |
| SO_0101 | fdnG | nitrate-inducible formate dehydrogenase molybdopterin-binding subunit FdnG | 4Fe-4S | 0.54 | 0.076 |
| SO_0102 | fdnH | nitrate-inducible formate dehydrogenase iron-sulfur subunit FdnH | 4Fe-4S | 0.44 | |
| SO_0103 | fdnI | nitrate-inducible formate dehydrogenase cytochrome b subunit FdnI | heme | 0.38 | 0.003 |
| SO_0104 | fdhE | nitrate-inducible formate dehydrogenase chaperone FdhE | Fe-S | 0.62 | 0.018 |
| SO_0139 | ftn | ferritin Ftn | iron binding | 1.60 | 0.066 |
| SO_0180 | PhyH | phytanoyl-CoA dioxygenase PhyH | iron-binding | ND | |
| SO_0264 | scyA | periplasmic monoheme cytochrome c5 ScyA | cytochrome c | 0.28 | 0.033 |
| SO_0266 | ccmF | cytochrome c synthetase cytochrome b containing quinol-haem oxidoreductase subunit CcmF | heme | 1.03 | 0.022 |
| SO_0292 | rpe | ribulose-phosphate 3-epimerase Rpe | mononuclear | 1.10 | 0.022 |
| SO_0311 | ygiW | uncharacterized radical SAM protein YgiQ | 4Fe-4S | ND | 0.031 |
| SO_0343 | acnD | 2-methyl citrate dehydratase Fe-S dependent AcnD | Fe-S | 1.08 | 0.006 |
| SO_0396 | SO_0396 | quinol:fumarate reductase menaquinol-oxidizing subunit FrdC | Fe-S | ND | 0.029 |
| SO_0397 | SO_0397 | quinol:fumarate reductase menaquinol-oxidizing subunit FrdC | Fe-S | ND | |
| SO_0399 | frdB | quinol:fumarate reductase FeS subunit FrdB | 2Fe-2S | 0.62 | 0.977 |
| SO_0432 | acnB | aconitate hydratase AcnB | 4Fe-4S | 0.82 | 0.005 |
| SO_0478 | sirE | cytochrome c maturation system haem lyase subunit SirE | heme | ND | |
| SO_0479 | SirA | sulfite reductase octaheme cytochrome c SirA | cytochrome c | ND | |
| SO_0483 | sirC | 4Fe-4S ferredoxin SirC | 4Fe-4S | 0.60 | 0.013 |
| SO_0528 | SO_0528 | radical SAM protein | 4Fe-4S | 1.06 | 0.031 |
| SO_0608 | petA | ubiquinol-cytochrome c reductase FeS subunit PetA | Fe-S | 0.72 | 0.019 |
| SO_0609 | petB | ubiquinol-cytochrome c reductase cytochrome b subunit PetB | heme | 0.78 | 0.074 |
| SO_0610 | petC | ubiquinol-cytochrome c reductase cytochrome c1 subunit PetC | cytochrome c | 0.70 | 0.036 |
| SO_0714 | SO_0714 | periplasmic monoheme cytochrome c4 | cytochrome c | ND | |
| SO_0716 | SorB | sulfite dehydrogenase cytochrome c subunit SorB | cytochrome c | ND | |

| | | | | | |
|---|---|---|---|---|---|
| SO_0717 | SO_0717 | periplasmic monoheme cytochrome c4 | cytochrome c | ND | |
| SO_0725 | katG2 | catalase/peroxidase HPI KatG-1 | heme | 2.26 | 0.031 |
| SO_0740 | SO_0740 | Dyp-type heme-dependent peroxidase | heme | 0.94 | 0.011 |
| SO_0744 | fbpA | ABC-type Fe3+ uptake system substrate-binding component FbpA | iron binding | 2.04 | 0.007 |
| SO_0747 | fpr | ferredoxin--NADP reductase Fpr | 2Fe-2S | 1.23 | 0.017 |
| SO_0750 | glsF | ferredoxin-dependent glutamate synthase GlsF | 3Fe-4S | 1.00 | 0.025 |
| SO_0845 | napB | periplasmic nitrate reductase cytochrome c subunit NapB | cytochrome c | 1.10 | 0.049 |
| SO_0848 | napA | periplasmic nitrate reductase molybdopterin-binding subunit NapA | Fe-S | 1.14 | 0.026 |
| SO_0849 | napD | periplasmic nitrate reductase chaperone NapD | 4Fe-4S | 0.84 | 0.090 |
| SO_0939 | SO_0939 | split-soret diheme cytochrome c | cytochrome c | ND | |
| SO_0944 | SO_0944 | radical SAM protein TatD family-associated | 4Fe-4S | ND | 0.982 |
| SO_0970 | fccA | Flavocytochrome c | cytochrome c | 0.34 | 0.086 |
| SO_0980 | rlmC | RNA methyltransferase, TrmA family | 4Fe-4S | 0.98 | 0.019 |
| SO_0988 | SO_0988 | molybdopterin-binding oxidoreductase | 2Fe-2S | 0.74 | 0.021 |
| SO_1014 | nuoI | NADH-ubiquinone oxidoreductase subunit I NuoI | 4Fe-4S | 0.46 | 0.022 |
| SO_1016 | nuoG | NADH-ubiquinone oxidoreductase subunit G NuoG | Fe-S | 0.47 | |
| SO_1017 | nuoF | NADH-ubiquinone oxidoreductase subunit F NuoF | Fe-S | 1.08 | 0.025 |
| SO_1019 | nuoC | NADH dehydrogenase I, C/D subunits | Fe-S | 1.00 | 0.062 |
| SO_1020 | nuoB | NADH-ubiquinone oxidoreductase subunit B NuoB | Fe-S | 1.01 | 0.047 |
| SO_1062 | def2 | polypeptide deformylase | mononuclear | 0.95 | 0.039 |
| SO_1070 | katB | catalase HPII KatB | heme | 2.49 | 0.014 |
| SO_1098 | SO_1098 | 2OG-Fe(II) oxygenase family protein | mononuclear | 1.12 | 0.014 |
| SO_1101 | luxS | S-ribosylhomocysteinase LuxS | iron-binding | 1.05 | 0.013 |
| SO_1111 | brf2 | bacterioferritin subunit 2 Bfr2 | iron binding | 0.58 | 0.270 |
| SO_1112 | brf1 | bacterioferritin subunit 1 Bfr1 | iron binding | 0.54 | 0.001 |
| SO_1158 | dpsA | DNA-binding ferritin-like protein (oxidative damage protectant) DpsA | iron binding | 1.53 | 0.104 |
| SO_1161 | lipA | lipoic acid synthetase LipA | Fe-S | 1.01 | 0.038 |

| | | | | | |
|---|---|---|---|---|---|
| SO_1181 | miaB | tRNA-N(6)-(isopentenyl)adenosine-37 thiotransferase enzyme MiaB | 4Fe-4S | 0.94 | 0.022 |
| SO_1181 | miaB | tRNA-N(6)-(isopentenyl)adenosine-37 thiotransferase enzyme MiaB | Fe-S | 0.94 | 0.879 |
| SO_1233 | torC | trimethylamine N-oxide reductase membrane anchored cytochrome c component TorC | cytochrome c | ND | |
| SO_1251 | SO_1251 | ferredoxin 4Fe-4S | 4Fe-4S | ND | 1.127 |
| SO_1304 | erpA | iron-sulfur cluster assembly insertion protein ErpA | Fe-S | 1.08 | 0.085 |
| SO_1324 | gltD | NADPH-dependent glutamate synthase small subunit GltD | Fe-S | 0.67 | 0.087 |
| SO_1325 | gltB | NADPH-dependent glutamate synthase large subunit GltB | 3Fe-4S | 0.54 | 0.033 |
| SO_1326 | SO_1326 | radical SAM superfamily protein YhcC | 4Fe-4S | ND | 1.092 |
| SO_1326 | SO_1326 | radical SAM superfamily protein YhcC | Fe-S | ND | 0.043 |
| SO_1363 | hcp | hydroxylamine reductase Hcp | Fe-S | ND | 0.052 |
| SO_1364 | SO_1364 | NADH oxidoreductase Hcr | 2Fe-2S | ND | 0.058 |
| SO_1413 | SO_1413 | flavocytochrome c heme submit | cytochrome c | ND | |
| SO_1421 | SO_1421 | periplasmic tetraheme flavocytochrome IfcA | cytochrome c | ND | |
| SO_1427 | dmsC | periplasmic decaheme cytochrome c DmsE | cytochrome c | ND | |
| SO_1429 | dmsA | extracellular dimethyl sulfoxide/manganese oxide reductase molybdopterin-binding subunit DmsA | Fe-S | 0.24 | 0.045 |
| SO_1430 | dmsB | extracellular dimethyl sulfoxide/manganese oxide reductase ferredoxin subunit DmsB | 4Fe-4S | 0.28 | 0.032 |
| SO_1490 | adhB | alcohol dehydrogenase II AdhB | iron-binding | 0.68 | 1.518 |
| SO_1519 | lldF | L-lactate dehydrogenase iron-sulfur cluster-binding protein LldF | Fe-S | 0.88 | 0.015 |
| SO_1521 | dld | respiratory FAD-dependent D-lactate dehydrogenase Dld | 4Fe-4S | 0.70 | |
| SO_1571 | SO_1571 | bifunctional DNA-binding protein / oxidoreductase | Fe-S | 1.36 | 0.076 |
| SO_1659 | SO_1659 | surface localized decaheme cytochrome c lipoprotein | cytochrome c | ND | |
| SO_1663 | napF | nitrate reductase (NapA) maturase NapF | Fe-S | 1.10 | 0.012 |
| SO_1666 | phhA | phenylalanine-4-hydroxylase PhhA | iron-binding | 1.28 | 0.042 |
| SO_1748 | SO_1748 | monoheme cytochrome c | cytochrome c | ND | |
| SO_1777 | mtrA | extracelllular iron oxide respiratory system periplasmic decaheme cytochrome c component MtrA | cytochrome c | 0.51 | 0.108 |
| SO_1778 | mtrC | extracellular iron oxide respiratory system surface decaheme cytochrome c component MtrC | cytochrome c | 0.31 | 0.044 |
| SO_1779 | omcA | extracelllular iron oxide respiratory system surface decaheme cytochrome c component OmcA | cytochrome c | 0.25 | 0.030 |

| | | | | | |
|---|---|---|---|---|---|
| SO_1780 | mtrF | extracellular respiratory system surface decaheme cytochrome c component MtrF | cytochrome c | 1.09 | 0.018 |
| SO_1782 | mtrD | extracellular respiratory system periplasmic decaheme cytochrome c component MtrD | cytochrome c | ND | |
| SO_1783 | feoA | ferrous iron transport protein A FeoA | iron binding | 1.27 | 0.118 |
| SO_1788 | miaE | tRNA-(MS(2)IO(6)A)-hydroxylase MiaE | iron-binding | 0.98 | 0.091 |
| SO_1909 | SO_1909 | catalytic subunit of aromatic ring-opening dioxygenase | iron binding | 1.01 | 0.009 |
| SO_1927 | sdhC | succinate dehydrogenase cytochrome b556 subunit SdhC | heme | 0.66 | 0.061 |
| SO_1929 | sdhB | succinate dehydrogenase iron-sulfur protein SdhB | 4Fe-4S | 0.89 | 0.017 |
| SO_1937 | fur | transcriptional repressor of iron homeostasis Fur | iron binding | 0.12 | 0.057 |
| SO_1944 | SO_1944 | 2OG-Fe(II) oxygenase family protein | mononuclear | 1.12 | 0.014 |
| SO_1962 | hppD | 4-hydroxyphenylpyruvate dioxygenase HppD | iron-binding | 1.07 | 0.034 |
| SO_2043 | YedZ | oxidoreductase cytochrome b subunit YedZ | heme | ND | |
| SO_2097 | HyaC | periplasmic [Ni-Fe] hydrogenase cytochrome b subunit HyaC | heme | ND | |
| SO_2098 | hyaB | periplasmic | iron-binding | 0.69 | 0.042 |
| SO_2099 | hyaA | periplasmic | Fe-S | ND | 0.012 |
| SO_2144 | SO_2144 | NO-binding heme-dependent sensor protein | heme | 1.13 | 0.066 |
| SO_2178 | ccpA | diheme cytochrome c5 peroxidase CcpA | cytochrome c | 1.63 | 0.053 |
| SO_2222 | fumB | anaerobic fumarate hydratase FumB | Fe-S | 0.48 | 1.823 |
| SO_2248 | sdaA | L-serine ammonia-lyase SdaA | Fe-S | 0.82 | |
| SO_2263 | iscR | transcriptional repressor of iron-sulfur cluster assembly genes IscR | Fe-S | 1.03 | 0.040 |
| SO_2264 | iscS | cysteine desulfurase IscS | 2Fe-2S | 0.92 | 1.086 |
| SO_2265 | iscU | FeS cluster assembly scaffold protein IscU | Fe-S | 0.98 | 1.064 |
| SO_2266 | iscA | FeS cluster assembly acessory protein IscA | Fe-S | 0.93 | 0.073 |
| SO_2267 | hscB | co-chaperone Hsc20 HscB | Fe-S | 0.97 | 0.043 |
| SO_2268 | hscA | chaperone protein HscA | Fe-S | 1.06 | 0.837 |
| SO_2269 | fdx | ferredoxin 2Fe-2S type ISC system Fdx | Fe-S | 0.91 | 0.055 |
| SO_2271 | iscX | FeS assembly protein IscX | iron-binding | 1.13 | 0.174 |
| SO_2342 | nadA | quinolinate synthetase NadA | 4Fe-4S | 1.11 | |

| | | | | | |
|---|---|---|---|---|---|
| SO_2356 | fnr | oxygen-responsive transcriptional regulator of anaerobiosis response Fnr | Fe-S | 0.99 | 0.004 |
| SO_2361 | ccoP | Cbb3-type cytochrome c oxidase subunit III CcoP | cytochrome c | 0.89 | 0.063 |
| SO_2363 | ccoO | Cbb3-type cytochrome c oxidase subunit II CcoO | cytochrome c | 0.82 | 0.016 |
| SO_2364 | CcoN | Cbb3-type cytochrome c oxidase subunit I CcoN | heme | 0.83 | 0.005 |
| SO_2408 | SO_2408 | glycine radical enzyme activase YjjW family | 4Fe-4S | ND | 0.658 |
| SO_2416 | nrdB | aerobic ribonucleoside-diphosphate reductase beta subunit NrdB | binuclear | 1.08 | 0.014 |
| SO_2417 | yfaE | ferredoxin cofactor maintenance protein YfaE | Fe-S | ND | 0.060 |
| SO_2419 | fadH | NADP-dependent 24-dienoyl-CoA reductase FadH | Fe-S | 0.73 | 0.030 |
| SO_2440 | SO_2440 | thiazole biosynthesis protein ThiH | 4Fe-4S | ND | 0.033 |
| SO_2477 | kdnB | alcohol dehydrogenase, iron-containing | iron-binding | 0.85 | 1.219 |
| SO_2501 | queE | queuosine biosynthesis radical activating enzyme QueE | Fe-S | 1.06 | 0.043 |
| SO_2508 | rnfA | ion (H+ or Na+)-translocating NADH:ferredoxin oxidoreductase subunit RnfA | Fe-S | ND | |
| SO_2509 | rnfB | ion (H+ or Na+)-translocating NADH:ferredoxin oxidoreductase subunit RnfB | Fe-S | 1.02 | 1.068 |
| SO_2510 | rnfC | ion (H+ or Na+)-translocating NADH:ferredoxin oxidoreductase subunit RnfC | Fe-S | 1.02 | |
| SO_2514 | nth | endonuclease III Nth | 4Fe-4S | 1.48 | 0.957 |
| SO_2530 | def1 | polypeptide deformylase | mononuclear | 1.091 | |
| SO_2589 | SO_2589 | 2OG-Fe(II) oxygenase family protein | iron-binding | 1.12 | 0.014 |
| SO_2618 | apbC | scaffold protein for | 4Fe-4S | 1.10 | 0.945 |
| SO_2643 | SO_2643 | oxidoreductase FAD-binding protein | Fe-S | 1.15 | 0.007 |
| SO_2726 | SO_2726 | cytochrome b | heme | 0.54 | 0.006 |
| SO_2727 | cctA | periplasmic tetraheme cytochrome c CctA | cytochrome c | 0.35 | 0.001 |
| SO_2740 | bioB | biotin synthase BioB | Fe-S | 0.75 | |
| SO_2805 | NnrS | heme-copper-containing inner membrane protein NnrS | heme | ND | |
| SO_2833 | nrdG | anaerobic ribonucleoside-triphosphate reductase activating protein NrdG | 4Fe-4S | ND | 0.021 |
| SO_2881 | sodB | Fe/Mn superoxide dismutase SodB | iron-binding | 0.50 | 0.014 |
| SO_2913 | pflA | pyruvate formate-lyase 1 activating enzyme PflA | 4Fe-4S | 0.99 | 0.025 |
| SO_2930 | SO_2930 | bifunctional pectinolytic enzyme/cytochrome c | cytochrome c | ND | |

| | | | | | |
|---|---|---|---|---|---|
| SO_2931 | SO_2931 | cytochrome c lipoprotein | cytochrome c | ND | |
| SO_3034 | putB | ferric putrebactin reductase PutB | 2Fe-2S | ND | |
| SO_3048 | SO_3048 | aldehyde oxidase family molybdopterin cytosine dinucleotide-binding subunit | 2Fe-2S | 0.63 | |
| SO_3049 | SO_3049 | aldehyde oxidase / xanthine dehydrogenase family FeS subunit | 2Fe-2S | 0.60 | |
| SO_3056 | SO_3056 | flavocytochrome c heme submit | cytochrome c | ND | |
| SO_3285 | cydB | cytochrome d ubiquinol oxidase subunit II CydB | heme | 0.84 | 0.006 |
| SO_3286 | cydA | cytochrome d ubiquinol oxidase subunit I CydA | heme | 0.79 | 0.008 |
| SO_3300 | SO_3300 | flavocytochrome c heme submit | cytochrome c | ND | |
| SO_3312 | ispG | 4-hydroxy-3-methylbut-2-en-1-yl diphosphate synthase IspG | 4Fe-4S | 1.03 | |
| SO_3315 | rlmN | 23S rRNA (adenine2503-C2)-methyltransferase RlmN | Fe-S | 0.91 | 0.650 |
| SO_3315 | rlmN | 23S rRNA (adenine2503-C2)-methyltransferase RlmN | Fe-S | 1.09 | |
| SO_3359 | yggW | radical SAM superfamily protein YggW | 4Fe-4S | 1.00 | 0.016 |
| SO_3371 | ycfB | cytochrome B561 YceJ | heme | 0.89 | 0.203 |
| SO_3420 | SO_3420 | monoheme cytochrome c | cytochrome c | 0.61 | 0.015 |
| SO_3421 | SO_3421 | cytochrome b561 | heme | 1.06 | 0.014 |
| SO_3529 | ispH | 1-hydroxy-2-methyl-2-(E)-butenyl 4-diphosphate reductase IspH | Fe-S | 0.97 | |
| SO_3549 | SO_3549 | hemerythrin | binuclear | 0.87 | 0.041 |
| SO_3563 | SO_3563 | 2OG-Fe(II) oxygenase family protein | mononuclear | 1.01 | 0.132 |
| SO_3596 | SO_3596 | protein of unknown function DUF1289 | Fe-S | ND | |
| SO_3623 | SO_3623 | flavocytochrome c heme submit | cytochrome c | ND | |
| SO_3662 | SO_3662 | inner membrane 4Fe-4S ferredoxin | 4Fe-4S | 1.29 | 0.020 |
| SO_3719 | SO_3719 | cytochrome b561 | heme | ND | |
| SO_3737 | cysI | sulfite reductase (NADPH) siroheme FeS subunit CysI | 4Fe-4S | 0.70 | |
| SO_3861 | queG | 4Fe-4S binding protein YjeS | 4Fe-4S | 0.92 | 0.056 |
| SO_3890 | SO_3890 | energy taxis-modulating methyl accepting sensory transducer with hemerythrin sensory domain | iron-binding | 1.25 | 0.040 |
| SO_3913 | SO_3913 | Fe2+-dependent dioxygenase | iron-binding | 4.42 | 0.119 |
| SO_3920 | hydA | periplasmic [Fe-Fe] hydrogenase large subunit | iron-binding | 0.61 | 0.006 |

| | | | | | |
|---|---|---|---|---|---|
| SO_3922 | fdh | formate dehydrogenase cytochrome b Fdh | heme | ND | |
| SO_3923 | hydG | Fe hydrogenase maturation rSAM protein HydG | Fe-S | 1.06 | 1.004 |
| SO_3925 | hydE | Fe hydrogenase maturation rSAM protein HydE | Fe-S | ND | 0.044 |
| SO_3926 | hydF | Fe hydrogenase maturation GTPase HydF | iron-binding | 1.16 | 0.101 |
| SO_3980 | nrfA | ammonia-forming nitrite reductase NrfA | cytochrome c | 0.79 | 0.792 |
| SO_4047 | SO_4047 | SoxA-like diheme cytochrome c | cytochrome c | 0.54 | 0.085 |
| SO_4048 | SO_4048 | diheme cytochrome c4 | cytochrome c | 0.49 | 0.014 |
| SO_4061 | phsB | sulfur reductase FeS subunit PhsB | 4Fe-4S | 0.19 | 0.089 |
| SO_4062 | phsA | sulfur reductase molybdoperterin-binding subunit PhsA | 4Fe-4S | 0.52 | 0.095 |
| SO_4072 | rimO | ribosomal protein S12 methylthiotransferase RimO | 4Fe-4S | 0.84 | 0.019 |
| SO_4072 | rimO | ribosomal protein S12 methylthiotransferase RimO | 4Fe-4S | 1.09 | |
| SO_4142 | SO_4142 | periplasmic monoheme cytochrome c | cytochrome c | ND | |
| SO_4144 | SO_4144 | octaheme tetrathionate reductase Otr | cytochrome c | ND | |
| SO_4234 | leuC | 3-isopropylmalate dehydratase large subunit LeuC | 4Fe-4S | 0.69 | 0.037 |
| SO_4311 | cyaY | iron donor for FeS cluster assembly CyaY | iron binding | 1.03 | 0.034 |
| SO_4345 | ilvD | dihydroxy-acid dehydratase IlvD | 2Fe-2S | 0.96 | 0.742 |
| SO_4354 | SO_4354 | protein of unknown function UPF0153 | iron-binding | 0.91 | 0.103 |
| SO_4357 | SO_4357 | extracellular oxidoreductase FeS binding subunit | 4Fe-4S | ND | 0.827 |
| SO_4358 | SO_4358 | extracellular oxidoreductase molybdopterin-binding lipoprotein subunit | 4Fe-4S | 1.09 | 0.062 |
| SO_4360 | SO_4360 | periplasmic decaheme cytochrome c MtrA family | cytochrome c | ND | |
| SO_4404 | SO_4404 | iron-sulfur cluster-binding protein | 4Fe-4S | ND | 0.064 |
| SO_4405 | katG1 | bifunctional catalase/peroxidase HPI KatG | heme | 1.29 | 0.325 |
| SO_4452 | moaA | molybdenum cofactor biosynthesis protein A | 4Fe-4S | 0.96 | |
| SO_4453 | etfQ | electron transfer flavoprotein-ubiquinone oxidoreductase EtfQ | 4Fe-4S | 1.15 | 0.149 |
| SO_4463 | SO_4463 | 2OG-Fe(II) oxygenase family protein | mononuclear | ND | |
| SO_4469 | yqhD | NADP-dependent alcohol dehydrogenase iron-containing YqhD | iron-binding | 1.02 | 0.065 |
| SO_4483 | SO_4483 | cytochrome b | heme | ND | |

| | | | | | |
|---|---|---|---|---|---|
| SO_4484 | SO_4484 | monoheme cytochrome c Shp | cytochrome c | ND | |
| SO_4485 | SO_4485 | diheme cytochrome c Dhc | cytochrome c | 1.15 | 0.054 |
| SO_4506 | SO_4506 | iron-sulfur cluster-binding protein | Fe-S | 0.47 | 1.619 |
| SO_4509 | fdhA | formate dehydrogenase, alpha subunit | Fe-S | 0.96 | 0.060 |
| SO_4510 | fdhB | formate dehydrogenase, iron-sulfur subunit | Fe-S | 1.32 | 1.095 |
| SO_4513 | fdhA | Fnr-inducilble formate dehydrogenase molybdopterin-binding subunit FdhA | 4Fe-4S | 0.85 | |
| SO_4514 | fdhB | Fnr-inducible formate dehydrogenase FeS subunit FdhB | 4Fe-4S | 0.60 | 0.093 |
| SO_4515 | fdhC | Fnr-inducible formate dehydrogenase cytochrome b subunit FdhC | heme | ND | |
| SO_4520 | SO_4520 | coproporphyrinogen III oxidase oxygen-independent | 4Fe-4S | 0.49 | 0.011 |
| SO_4572 | SO_4572 | triheme cytochrome c | cytochrome c | ND | |
| SO_4591 | cymA | membrane anchored tetraheme cytochrome c CymA | cytochrome c | 0.56 | 0.059 |
| SO_4606 | cyoA | aa3-type cytochrome c oxidase subunit II CoxB | cytochrome c | ND | |
| SO_4607 | CoxA | aa3 type cytochrome c oxidase subunit I CoxA | heme | ND | |
| SO_4619 | nfuA | iron-sulfur cluster biogenesis scaffold protein NfuA | 4Fe-4S | 0.90 | 0.039 |
| SO_4666 | cytB | diheme cytochrome c4 CytcB | cytochrome c | 0.55 | 0.061 |
| SO_4673 | tdh | L-threonine 3-dehydrogenase Tdh | mononuclear | 1.03 | |
| SO_4724 | moaA | tungsten cofactor biosynthesis protein MoaA | 4Fe-4S | 1.08 | 0.020 |
| SO_4730 | hemN | coproporphyrinogen III oxidase oxygen-independent HemN | Fe-S | 0.69 | 0.025 |
| SO_4737 | ccoG | Cbb3-type cytochrome oxidase assembly protein CcoG | 4Fe-4S | 0.98 | 0.716 |
| SO_4806 | SO_4806 | 2OG-Fe(II) oxygenase family protein | mononuclear | 1.12 | 0.014 |
